# Dual role of HDAC10 in lysosomal exocytosis and DNA repair promotes neuroblastoma chemoresistance

**DOI:** 10.1038/s41598-018-28265-5

**Published:** 2018-07-03

**Authors:** Johannes Ridinger, Emily Koeneke, Fiona R. Kolbinger, Katharina Koerholz, Siavosh Mahboobi, Lars Hellweg, Nikolas Gunkel, Aubry K. Miller, Heike Peterziel, Peter Schmezer, Anne Hamacher-Brady, Olaf Witt, Ina Oehme

**Affiliations:** 1 Preclinical Program, Hopp Children’s Cancer Center at NCT Heidelberg (KiTZ), Heidelberg, Germany; 2 Clinical Cooperation Unit Pediatric Oncology, German Cancer Research Center (DKFZ), and German Cancer Consortium (DKTK), Im Neuenheimer Feld 280, 69120 Heidelberg, Germany; 3 Faculty of Biosciences, University of Heidelberg, Heidelberg, Germany; 4 University of Heidelberg, Heidelberg, Germany; 5 Institute of Pharmacy, University of Regensburg, Regensburg, Germany; 6 Research Group Cancer Drug Development, German Cancer Research Center, Heidelberg, Germany; 7 Division of Epigenomics and Cancer Risk Factors, German Cancer Research Center, Heidelberg, Germany; 8 Johns Hopkins University, Bloomberg School of Public Health, Baltimore, United States; 9 Department of Pediatric Oncology, Hematology and Immunology, University Hospital Heidelberg, Heidelberg, Germany

## Abstract

Drug resistance is a leading cause for treatment failure in many cancers, including neuroblastoma, the most common solid extracranial childhood malignancy. Previous studies from our lab indicate that histone deacetylase 10 (HDAC10) is important for the homeostasis of lysosomes, i.e. acidic vesicular organelles involved in the degradation of various biomolecules. Here, we show that depleting or inhibiting HDAC10 results in accumulation of lysosomes in chemotherapy-resistant neuroblastoma cell lines, as well as in the intracellular accumulation of the weakly basic chemotherapeutic doxorubicin within lysosomes. Interference with HDAC10 does not block doxorubicin efflux from cells via P-glycoprotein inhibition, but rather via inhibition of lysosomal exocytosis. In particular, intracellular doxorubicin does not remain trapped in lysosomes but also accumulates in the nucleus, where it promotes neuroblastoma cell death. Our data suggest that lysosomal exocytosis under doxorubicin treatment is important for cell survival and that inhibition of HDAC10 further induces DNA double-strand breaks (DSBs), providing additional mechanisms that sensitize neuroblastoma cells to doxorubicin. Taken together, we demonstrate that HDAC10 inhibition in combination with doxorubicin kills neuroblastoma, but not non-malignant cells, both by impeding drug efflux and enhancing DNA damage, providing a novel opportunity to target chemotherapy resistance.

## Introduction

Neuroblastoma is a pediatric tumor of the sympathetic nervous system and the most common extracranial solid tumor in childhood. Depending on the underlying tumor biology, its clinical presentation and the course of disease vary immensely, ranging from localized to highly aggressive disease (reviewed in^1,2^). This has profound impact on prognosis and therapy success, which remains poor in high-risk neuroblastoma patients despite the intensification of treatment regimens^3^ (reviewed in^1^).

Multidrug resistance (MDR) is a common problem in cancer treatment and a major cause for treatment failure in cancers, including neuroblastoma (reviewed in^4^). Mechanisms of neuroblastoma drug resistance include deregulation of apoptosis^5,6^ (reviewed in^7^), the presence of cancer stem cells^8^, alterations or mutations of drug targets^9^, increased DNA repair capacity^10^, as well as increased drug efflux. The latter has been strongly attributed to the presence and activity of multidrug resistance promoting, ATP-dependent drug efflux pumps such as P-glycoprotein (P-gp/MDR1)^11^ (reviewed in^12^). Cancer cells can further increase their drug resistance by exploiting stress resistance mechanisms like (macro-) autophagy (hereafter called autophagy), a lysosomal degradation pathway responsible for the degradation of aged organelles and proteins. Collective evidence indicates that autophagy can be induced by therapeutic agents, thereby facilitating cancer cell survival during drug induced metabolic stress (reviewed in^13^).

Recent evidence indicates that lysosomes play a crucial role in MDR. Hydrophobic weakly-basic chemotherapeutic drugs (including doxorubicin) can diffuse across both plasma membrane and lysosomal membranes. Due to their low pH, lysosomes are able to protonate and sequester these drugs, thereby preventing drugs from reaching their cellular target^14–16^ (reviewed in^17,18^). Moreover, lysosomes might provide an additional defense mechanism by clearing drugs from cells in a process called lysosomal exocytosis, in which lysosomes fuse with the plasma membrane, releasing their cargo to the extracellular space^19–21^ (reviewed in^22,23^).

Histone deacetylases (HDACs) make up a class of enzymes that catalyze the removal of acetyl groups from lysine residues of both nuclear (e.g. histones) and cytosolic proteins (reviewed in^24,25^). Given their involvement in numerous cancer-relevant processes, their good druggability and their involvement in important tumor-relevant pathways, HDACs are an attractive target for novel therapeutic approaches. HDAC inhibitors (HDACis) exert a variety of anti-tumorigenic effects, and a number of pan or broad-spectrum HDAC inhibitors are approved for cancer treatment (reviewed in^26^). Recently, HDACs and their inhibitors have also been shown to play a role in lysosomal biology, and class IIb family members (HDACs 6 and 10) have been repeatedly linked to cellular stress response, protein degradation, and autophagy^27–31^ (reviewed in^32^). In addition, HDAC10 plays a role in DNA repair^33,34^.

We have previously identified the class IIb HDAC member HDAC10 as a prognostic marker and druggable target in high-risk neuroblastoma, where it promotes late-stage autophagic flux and chemoresistance^31^ (reviewed in^35^).

Here, we further unravel the role of HDAC10 in lysosome-coupled mechanisms, such as lysosomal exocytosis, which plays a critical role in neuroblastoma resistance against doxorubicin. We demonstrate that targeting HDAC10 sensitizes neuroblastoma cells to doxorubicin by inhibiting drug efflux via lysosomal exocytosis and enhancing DNA double-strand breaks, thereby promoting tumor cell death in chemotherapy resistant neuroblastoma models.

## Results

### Depletion and inhibition of HDAC10, but not HDAC6, promotes the accumulation of lysosomes in neuroblastoma cells

**Figure 1:**
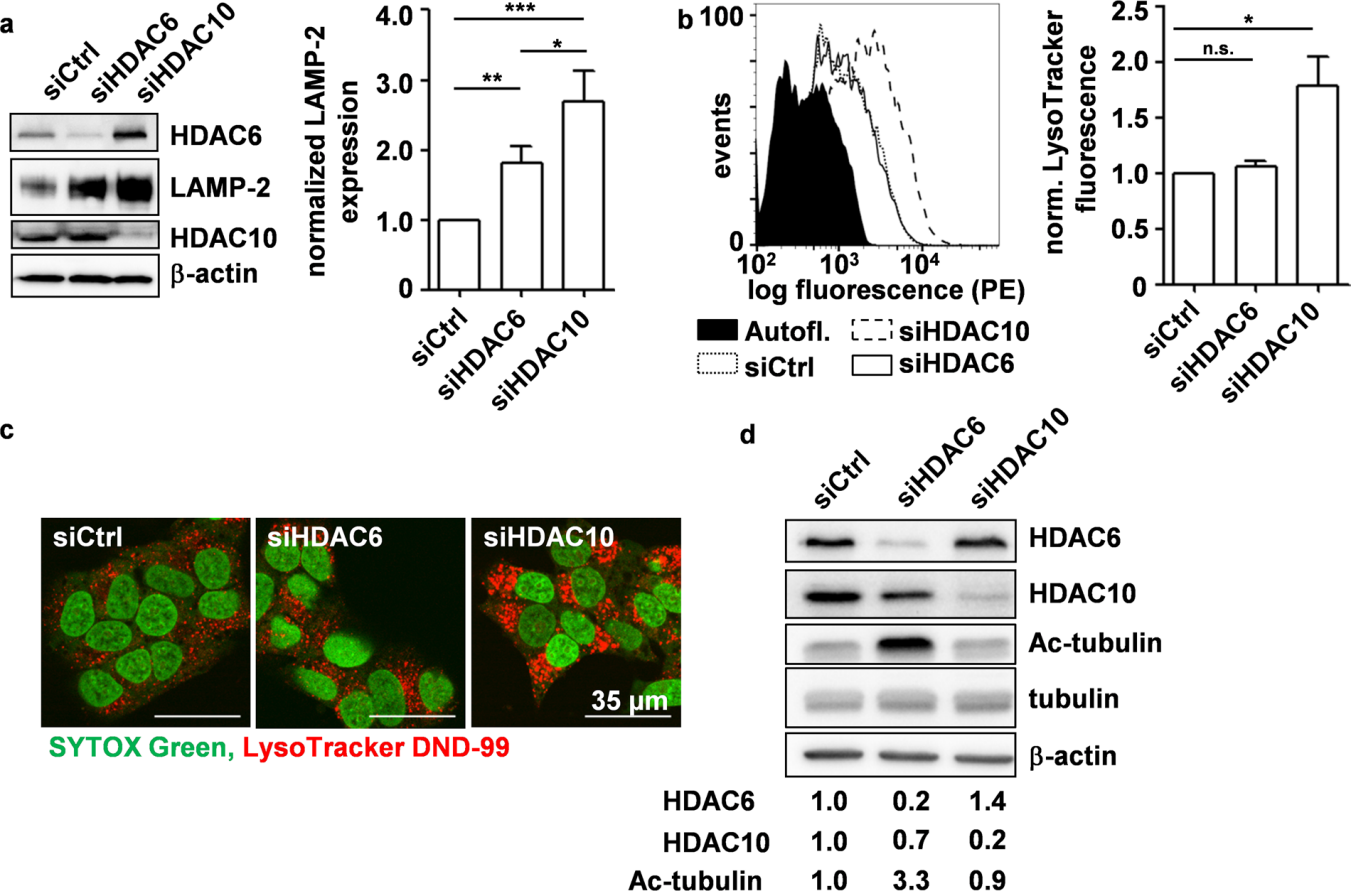
HDAC10 depletion promotes the accumulation of lysosomes in neuroblastoma cells. (**a**) Western blot analysis of LAMP-2 expression 6d after HDAC6/10 knockdown (left panel) including densitometric quantification of LAMP-2 expression of n = 4 experiments (right panel). Bands of indicated proteins were developed on the same blot (**b**) Flow cytometric analysis of LysoTracker DND-99 fluorescence in BE(2)-C neuroblastoma cells 6d after siRNA-mediated HDAC6/10 knockdown. Fluorescence histogram (left panel) and bar graph depicting normalized mean fluorescence of n = 4 experiments (right panel) are shown. (**c**) LysoTracker DND-99 staining 6d after transfection with siRNAs against HDAC6 and HDAC10, respectively. Nuclei were counterstained with SYTOX Green. (**d**) Western blot analysis of tubulin acetylation 72 h after knockdown of HDAC6 and HDAC10, respectively. Numbers below blot indicate HDAC6/10 expression normalized to β-actin and relative to siCtrl, as well as tubulin acetylation normalized to total tubulin and relative to siCtrl. Bands of indicated proteins were developed on the same blot. Statistical analyses were performed on non-normalized data using unpaired (**a**) or paired (**b**) two-tailed t-test (***p < 0.001; **0.001 ≤ p < 0.01; *0.01 ≤ p < 0.05). Error bars represent standard error of the mean (SEM).

Previous work of our lab has shown that interference with HDAC10 function leads to the accumulation of lysosomes and autophagolysosomes in neuroblastoma cell lines^31^. As various studies have pointed out roles for HDAC6 and HDAC10 in autophagosome-lysosome fusion, we compared the effects of HDAC6 and HDAC10 knockdown and inhibition on lysosomes via expression of lysosomal markers such as LAMP-2 on western blot, as well as via staining of lysosomes with the acidotropic LysoTracker DND-99 dye^30,31^. Western blot analysis of LAMP-2 expression after knockdown of HDAC6 or HDAC10 in highly chemoresistant *MYCN* amplified, *TP53* mutated SK-N-BE(2)-C neuroblastoma cells (hereafter referred to as BE(2)-C) revealed that both knockdown of HDAC6 and HDAC10 resulted in LAMP-2 accumulation, albeit to a significantly greater extent after HDAC10 knockdown (Fig. 1a). Expression of LAMP-2 alone is not sufficient for the quantification of lysosomes, as LAMP-2 is also found on late endosomes^36^. We therefore labeled BE(2)-C cells with LysoTracker and observed that RNAi-mediated depletion of HDAC10, but not HDAC6, substantially increased lysosomal staining and thus the accumulation of acidified lysosomes (Fig. 1b, Supplementary Fig. S1a). Enlargement of the lysosomal compartment was confirmed by fluorescence microscopic analysis of LysoTracker staining (Fig. 1c). Notably, lysosomal accumulation after HDAC10 knockdown was not restricted to the perinuclear cloud^37^, as lysosomes were distributed throughout the cytoplasm. Western blot analysis confirmed that only knockdown of HDAC6, but not HDAC10, increased tubulin K40 acetylation, showing that, despite their close structural relation, HDAC6 and HDAC10 have distinct cellular functions (Fig. 1d).

**Figure 2:**
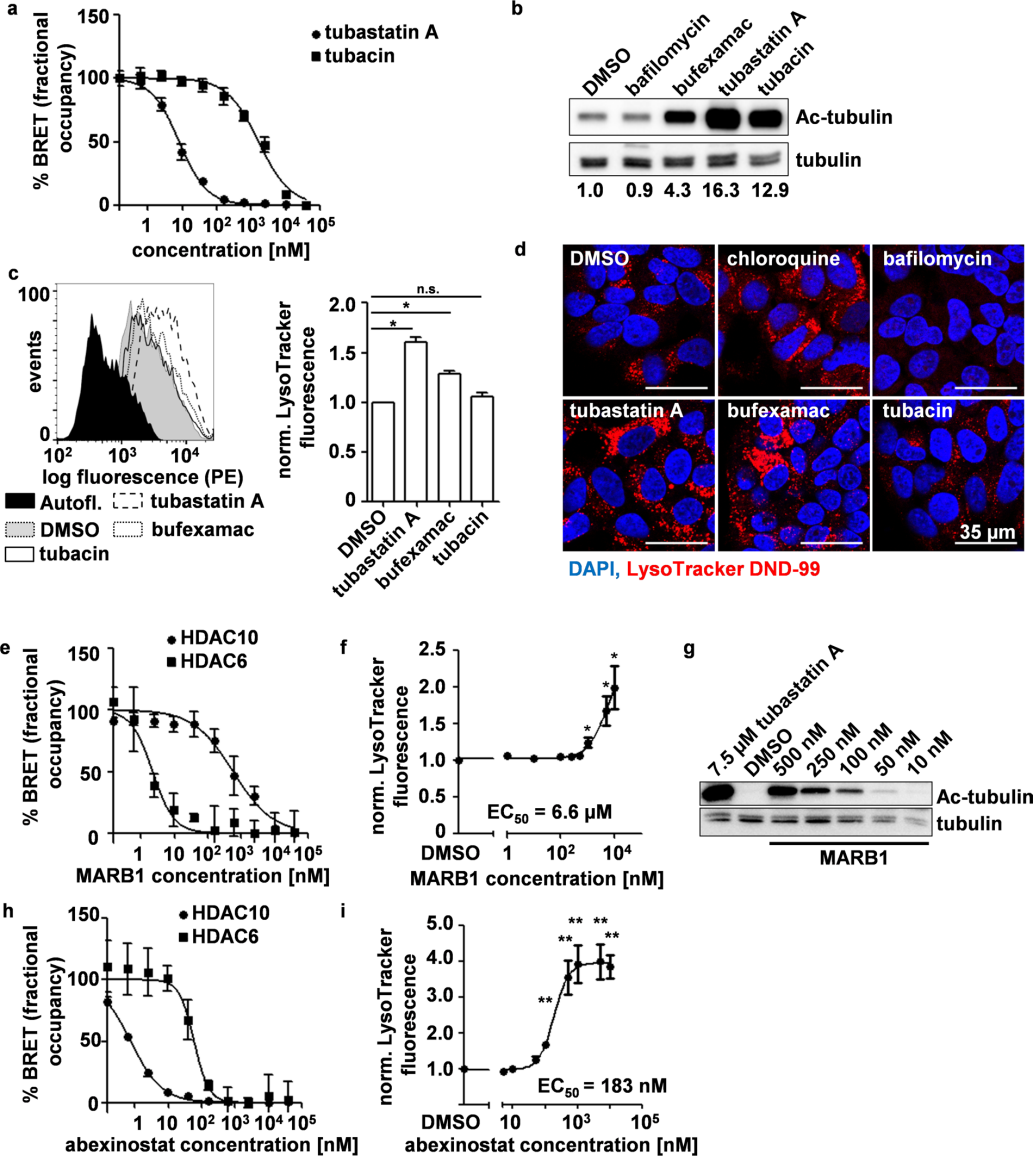
HDAC10 inhibition promotes the accumulation of lysosomes in neuroblastoma cells. (**a**) In cell target engagement assay (NanoBRET) analysis of HDAC10 interaction with HDAC6/10 inhibitor tubastatin A and HDAC6 inhibitor tubacin, respectively. Graphs depict mean amount of acceptor-occupied NanoLuc-HDAC10 relative to the total amount of NanoLuc-HDAC10 (% fractional occupancy, y-axis) versus logarithmic drug concentration (x-axis). (**b**) Western blot analysis of tubulin acetylation 24 h after treatment with bufexamac (30 µM), tubastatin A (7.5 µM) and tubacin (7.5 µM). Numbers below blot indicate tubulin acetylation normalized to total tubulin and relative to DMSO control. Tubulin and acetylated tubulin bands were derived from two equally loaded and simultaneously blotted gels. Equal transfer was checked by Ponceau S staining. (**c**) Flow cytometric analysis of LysoTracker DND-99 staining in BE(2)-C cells 24 h after treatment with HDAC6/10 inhibitors bufexamac (30 µM) and tubastatin A (7.5 µM) and HDAC6 inhibitor tubacin (7.5 µM). Fluorescence histogram (left panel including unstained cells (Autofl.)) and bar graph depicting normalized mean fluorescence of n = 4 experiments (right panel) are shown. (**d**) Fluorescence microscopy analysis of LysoTracker DND-99 staining 24 h after treatment with 30 µM bufexamac, 7.5 µM tubastatin A and 7.5 µM tubacin, lysosomal inhibitor (5 µM chloroquine) and vacuolar ATPase inhibitor (100 nM bafilomycin). Nuclei were counterstained with DAPI (4′,6-Diamidino-2-phenylindole). (**e**) NanoBRET analysis of HDAC6/10 interaction with Marbostat-100 (MARB1). Graphs depict mean amount of acceptor-occupied NanoLuc-HDAC6/10 relative to the total amount of NanoLuc-HDAC6/10 (% fractional occupancy, y-axis) versus logarithmic drug concentration (x-axis). (**f**) Flow cytometric analysis of LysoTracker DND-99 fluorescence 24 h after treatment with Marbostat-100 (MARB1). (**g**) Western blot analysis of tubulin acetylation 24 h after treatment with tubastatin A and Marbostat-100 (MARB1). Tubulin and acetylated tubulin bands were derived from two equally loaded membrane sections. Sections were separated by cutting after blotting. (**h**) NanoBRET analysis of HDAC6/10 interaction with abexinostat. (**i**) Flow cytometric analysis of LysoTracker DND-99 fluorescence 24 h after treatment with abexinostat. Graphs (**e**,**h**) depict mean LysoTracker fluorescence (y-axis) versus logarithmic concentration in nM (x-axis) of at least n = 4 experiments. Curves were fitted using non-linear curve fitting in GraphPad Prism version 5 (GraphPad Software Inc., San Diego, CA, USA). Statistical analyses were performed on non-normalized data using paired two-tailed t-test (***p < 0.001; **0.001 ≤ p < 0.01; *0.01 ≤ p < 0.05). Error bars represent standard error of the mean (SEM).

We next investigated if accumulation of lysosomes was also affected by inhibition of HDAC6 and HDAC10, respectively. Currently, there is no inhibitor available which selectively blocks HDAC10 activity. However, the HDAC6 inhibitors bufexamac^38^ and tubastatin A^31^ have been shown to be dual specific HDAC6/10 inhibitors, whereas tubacin is thought to be HDAC6 specific^39^. Since no bona fide downstream target protein of HDAC10 has been described to date, we performed an in-cell target engagement (NanoBRET) assay that measures competitive displacement of a fluorescently-labeled tracer from NanoLuc® Luciferase coupled HDAC10 enzyme. This assay confirmed that tubastatin A had strong affinity towards HDAC10 while tubacin did not (Fig. 2a). Western blot analysis of tubulin K40 acetylation as a marker for HDAC6 inhibition confirmed that bufexamac, tubastatin A and tubacin substantially inhibited HDAC6 at the concentrations used, with the dual inhibitor bufexamac displaying the lowest potency against HDAC6 (Fig. 2b). Flow cytometric analysis of LysoTracker staining after 24 h treatment revealed that HDAC6/10 inhibitors bufexamac and tubastatin A caused lysosomal accumulation while the HDAC6-specific inhibitor tubacin did not (Fig. 2c). Thus, lysosomal accumulation was not attributed to HDAC6 inhibitory capacity of the compounds at the indicated concentrations. Enlargement of the lysosomal compartment after use of HDAC6/10 inhibitors bufexamac and tubastatin A, but not HDAC6 inhibitor tubacin, was confirmed by fluorescence microscopy (Fig. 2d). Overall, together with our knockdown data, we concluded that lysosomal accumulation after HDAC6/10 inhibition was likely due to inhibition of HDAC10 function. To exclude that lysosomal accumulation after treatment with HDAC6/10 inhibitors was caused by off-target inhibition of class I HDACs, we treated BE(2)-C cells with 1 mM class I HDAC inhibitor valproic acid (VPA). VPA treatment, although substantially inhibiting HDACs 1–3 as shown by histone H3 acetylation, did not induce lysosomal accumulation (Supplementary Fig. S1b,c).

We also used the inhibitor Marbostat-100 (MARB1)^40,41^, which displayed strong HDAC6 inhibitory capacity in the nanomolar range (IC_50_ 2.21 nM), while inhibiting HDAC10 only at concentrations above 150 nM (IC_50_ 564 nM), as assessed by NanoBRET analysis (Fig. 2e). Accordingly, MARBOSTAT-100 increased LysoTracker staining only at concentrations above 1 µM (EC_50_ 6.6 µM) (Fig. 2f), while inducing tubulin acetylation at concentrations as little as 50 nM (Fig. 2g).

Abexinostat (PCI-24781) is a pan-HDAC inhibitor which is currently being used in phase I/II clinical trials. NanoBRET analysis revealed that abexinostat has strong HDAC10 inhibitory capacity (IC_50_ 0.74 nM) (Fig. 2h). Consistent with our findings above, abexinostat induced lysosomal accumulation at concentrations as low as 100 nM (EC_50_ 183 nM) (Fig. 2i).

### HDAC10 inhibition and depletion promote increased intracellular accumulation of doxorubicin

**Figure 3:**
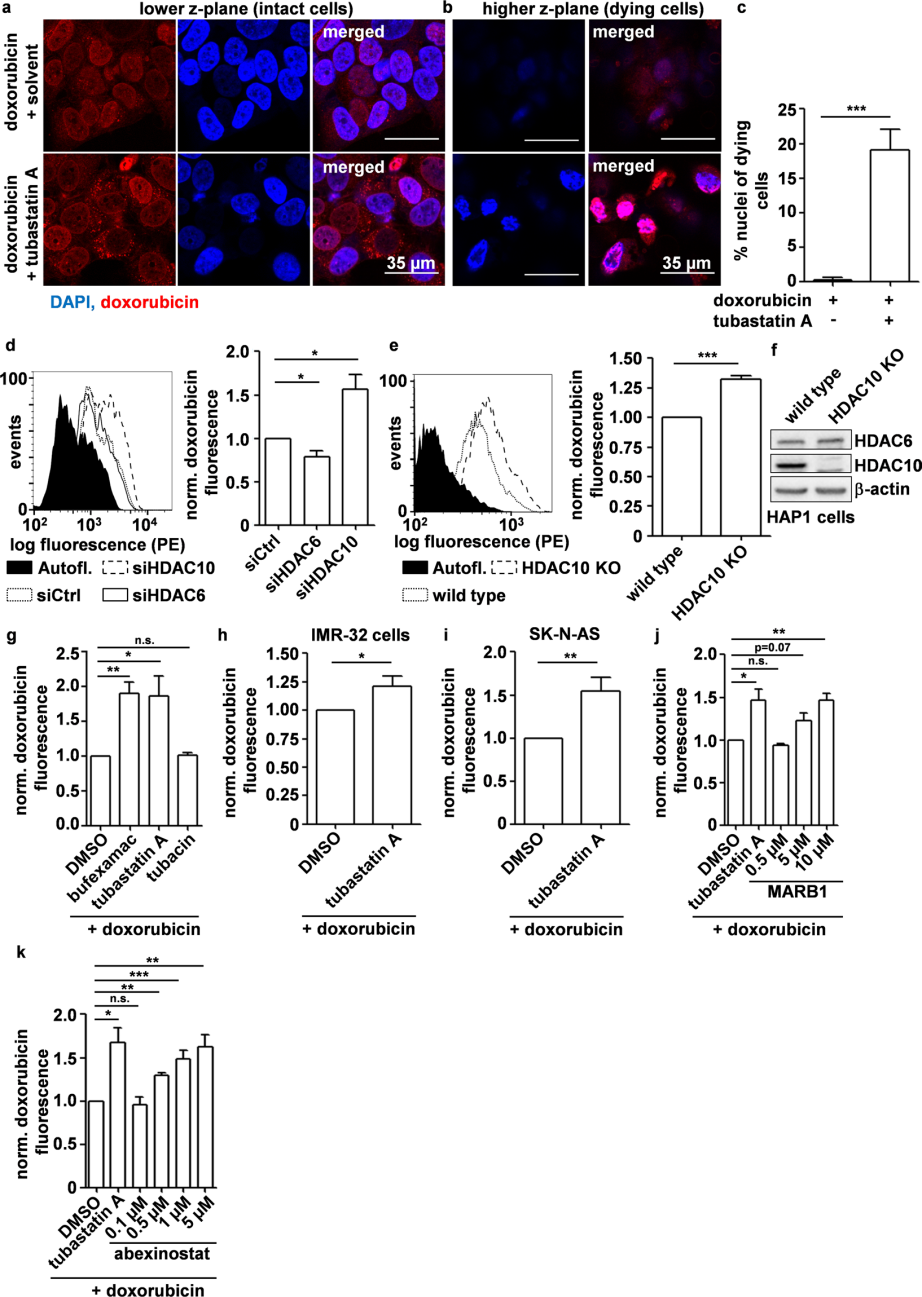
HDAC10 depletion and inhibition promote intracellular accumulation of doxorubicin. (**a**) Confocal fluorescence microscopy analysis of intracellular doxorubicin accumulation after 24 h treatment with 250 ng/ml doxorubicin +/− HDAC6/10 inhibitor (7.5 µM tubastatin A). Nuclei were counterstained with DAPI. Images in (**a**) and (**b**) are derived from corresponding z-stacks. (**a**) Depicts cells located in lower z-position. (**b**) Confocal fluorescence microscopy analysis of damaged, doxorubicin-loaded nuclei 24 h after doxorubicin treatment in higher z-plane of z-stacks described in (**a**). (**c**) Damaged nuclei were quantified manually and normalized to total nuclei per image (% damaged nuclei). Bar graph depicts mean % of damaged nuclei from n = 3 experiments. (**d,e**) Flow cytometric analysis of cellular doxorubicin fluorescence in BE(2)-C and HAP1 cells. (**d**) BE(2)-C cells were treated with 100 ng/ml doxorubicin for 24 h. Figure shows fluorescence histogram (left panel) and bar graph depicting mean fluorescence of n = 5 experiments normalized to cells transfected with control siRNA (right panel). (**e**) HAP1 wild type and HDAC10 knockout (HDAC10 KO) cells were treated with 25 ng/ml doxorubicin for 18 h. Figure shows fluorescence histogram (left panel) and bar graph depicting mean fluorescence of n = 6 experiments normalized to wild type cells (right panel). (**f**) Western Blot analysis of HDAC6 and HDAC10 expression in HAP1 wild type and HDAC10 KO cells. Bands of indicated proteins were developed on the same blot. (**g,h**) Flow cytometric quantification of cellular doxorubicin fluorescence. (**g**) Doxorubicin fluorescence in BE(2)-C cells after treatment with 100 ng/ml doxorubicin +/− HDAC6/10 inhibitors (30 µM bufexamac, 7.5 µM tubastatin A) and HDAC6 inhibitor tubacin (7.5 µM) for 48 h. Bar graph depicts mean fluorescence of n = 4 experiments normalized to DMSO control. (**h**,**i**) Doxorubicin fluorescence in IMR-32 cells (**h**) 18 h after treatment with 50 ng/ml doxorubicin +/− 7.5 µM tubastatin A (n = 5 experiments) and SK-N-AS cells (**i**) 48 h after treatment with 100 ng/ml doxorubicin +/− 7.5 µM tubastatin A (n = 4 experiments). (**j**,**k**) Doxorubicin fluorescence in BE(2)-C cells after 24 h treatment with 100 ng/ml doxorubicin +/− HDAC6/10 inhibitor Marbostat-100 (MARB1) (n = 4 experiments) (**j**) and pan HDAC inhibitor abexinostat (n = 5 experiments) (**k**). Statistical analyses were performed on non-normalized data using unpaired (**c**) or paired (**d**,**e**,**g**–**k**), two-tailed t-test (***p < 0.001; **0.001 ≤ p < 0.01; *0.01 ≤ p < 0.05). Error bars represent SEM.

Enlargement of the lysosomal compartment has been associated with increased sequestration of weakly basic chemotherapeutics and therapy resistance^14,16^. Our previous work demonstrated that HDAC6/10 inhibition increases sensitivity of neuroblastoma cell lines to therapeutically relevant weakly basic neuroblastoma chemotherapeutics such as doxorubicin^31^. We, therefore, analyzed the subcellular localization of doxorubicin in chemoresistant BE(2)-C cells in the presence or absence of tubastatin A via fluorescence microscopy, exploiting doxorubicin’s autofluorescent properties. Indeed, 24 h after treatment, doxorubicin was partly localized in perinuclear vesicles, and also in the nucleus (Fig. 3a). Doxorubicin presence in lysosomal vesicles was markedly stronger, and not restricted to perinuclear areas, when cells were co-treated with tubastatin A (Fig. 3a). Moreover, increased doxorubicin accumulation after addition of tubastatin A was not restricted to lysosomes but also evident in the nucleus. When acquiring z-stacks, we noticed that the combination of doxorubicin with HDAC6/10 inhibitor tubastatin A increased the number of dying cells displaying a highly condensed chromatin structure as compared to doxorubicin treatment alone. Notably, these nuclei of dying cells, which were only visible after combination with an HDAC6/10 inhibitor, were highly loaded with doxorubicin (Fig. 3a–c) and were located in a higher z-plane (Fig. 3b) than undamaged cells, which were found in lower z-positions of the corresponding z-stack (Fig. 3a). This suggested that intracellular accumulation, rather than strictly the lysosomal sequestration of doxorubicin, was enhanced by HDAC10 inhibition.

We next tested whether lysosomal perturbation by HDAC10 depletion or inhibition influenced intracellular levels of doxorubicin using flow cytometry. RNAi-mediated depletion of HDAC10 led to a roughly 1.5-fold increased mean fluorescence of intracellular doxorubicin 24 h after treatment with doxorubicin. In contrast, HDAC6 depletion slightly decreased the accumulation of doxorubicin in BE(2)-C neuroblastoma cells, again supporting a specific function of HDAC10 within class IIb HDACs (Fig. 3d). The effects following RNAi-mediated HDAC10 depletion could be confirmed using the commercially purchased near-haploid chronic myeloid leukemia (CML) HDAC10 knockout cell line (HAP1 HDAC10 KO), which accumulated slightly but significantly (p = 9.8 ∗ 10^−5^) higher amounts of doxorubicin than their HDAC10 expressing wild type counterpart (HAP1 wt) (Fig. 3e,f).

The finding that HDAC10 but not HDAC6 depletion increased doxorubicin accumulation in neuroblastoma cells further suggested that the increased doxorubicin accumulation depicted in Fig. 3a was mediated by tubastatin A’s ability to inhibit HDAC10^31^ rather than HDAC6. Measurement of intracellular accumulation of doxorubicin by co-treating BE(2)-C neuroblastoma cells with doxorubicin and bufexamac, tubastatin A or tubacin for 48 h revealed that addition of HDAC6/10 inhibitors bufexamac and tubastatin A increased intracellular doxorubicin (Fig. 3g). In contrast, 48 h co-treatment with HDAC6 specific inhibitor tubacin did not alter cellular doxorubicin content, again suggesting that doxorubicin accumulation was due to HDAC10 rather than HDAC6 inhibition. Similarly, HDAC6/10 inhibition increased accumulation of doxorubicin in two other neuroblastoma cell lines, IMR-32 (Fig. 3h) and SK-N-AS (Fig. 3i). In order to exclude that doxorubicin accumulation was caused by off-target effects due to long-term drug exposure, we treated BE(2)-C cells for 12 h with HDAC6/10 inhibitors before adding a higher dose of doxorubicin (1 µg/ml) for 3 h. Again, we observed that dual specific HDAC6/10 inhibitors tubastatin A and bufexamac increased intracellular doxorubicin levels while HDAC6 specific inhibitor tubacin did not (Supplementary Fig. S2).

Flow cytometric analysis of doxorubicin accumulation after co-treatment of BE(2)-C cells with doxorubicin and MARBOSTAT-100 increased intracellular doxorubicin levels only at concentrations where MARBOSTAT-100 strongly inhibited HDAC10 and induced lysosomal accumulation (5 µM and higher, Fig. 2f), while having no effect at lower concentrations which still clearly inhibited HDAC6 (Figs 2g and 3j).

Accumulation of doxorubicin in BE(2)-C cells was also achieved by co-treatment of BE(2)-C cells with the pan-HDAC inhibitor abexinostat, which has strong HDAC10 inhibitory activity. Abexinostat is currently being evaluated in phase I/II trials for combination treatment with doxorubicin^42,43^. Co-treatment of BE(2)-C cells with doxorubicin and abexinostat for 24 h increased intracellular doxorubicin amounts, when abexinostat was used at concentrations greater than or equal to 500 nM, where lysosomes robustly accumulated (Figs 2i and 3k).

In summary, depletion or inhibition of HDAC10, but not HDAC6 alone, increases the lysosomal compartment, associated with an increased intracellular accumulation of doxorubicin.

### HDAC6/10 inhibitors do not promote cellular doxorubicin accumulation by P-glycoprotein inhibition

**Figure 4:**
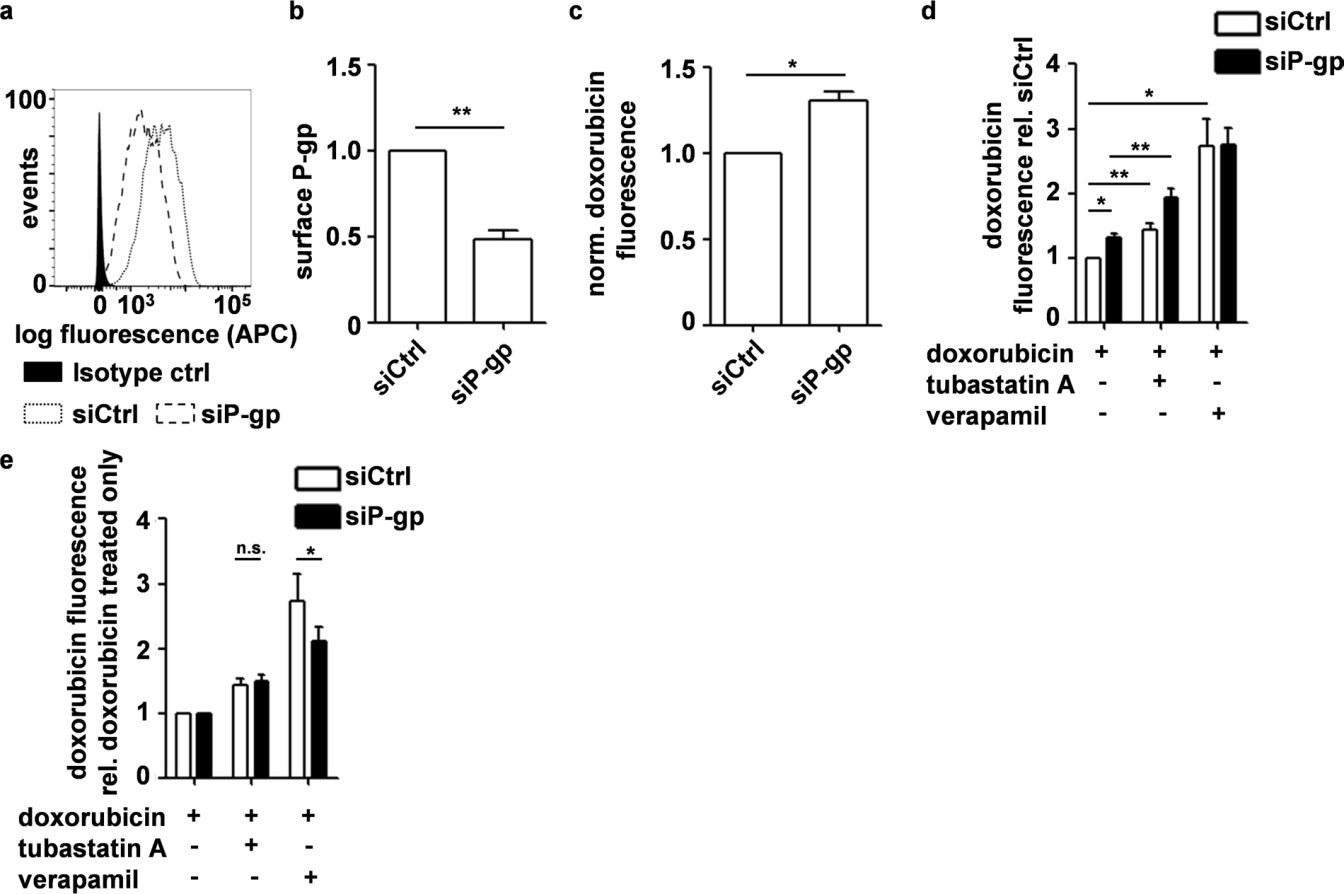
HDAC6/10 inhibition promotes cellular doxorubicin accumulation independently of P-glycoprotein (P-gp). (**a)** Histogram showing flow cytometric analysis of P-gp expression at the cell surface 5d after transfection with P-gp (siP-gp) and control siRNAs (siCtrl), respectively. (**b**) Flow cytometric quantification of cell surface P-gp expression normalized to siCtrl transfected cells 5d after siRNA transfection. Bar graph depicts normalized mean fluorescence of n = 5 experiments. (**c)** Flow cytometric quantification of doxorubicin in cells 24 h after beginning of treatment with 100 ng/ml doxorubicin and 5d after transfection with control and P-gp siRNA, respectively. Graph shows normalized fluorescence of n = 5 experiments. (**d)** Flow cytometric quantification of cellular doxorubicin 5d after siRNA transfection and 24 h after treatment with 100 ng/ml doxorubicin +/− 7.5 µM tubastatin A or 10 µM verapamil. Graph depicts mean fluorescence of n = 5 experiments normalized to doxorubicin fluorescence in siCtrl transfected cells treated with doxorubicin only. (**e)** Graph depicts data of (**d)** normalized to siCtrl and siP-gp transfected cells treated with doxorubicin only. Statistical analyses were performed on non-normalized data using paired, two-tailed t-test (***p < 0.001; **0.001 ≤ p < 0.01; *0.01 ≤ p < 0.05). Error bars represent SEM.

The ATP-binding cassette (ABC) transporter family of transmembrane proteins is known to promote the efflux and elimination of various chemotherapeutics from cancer cells. Members of this family, such as P-glycoprotein (P-gp), are known to promote doxorubicin secretion and provide doxorubicin resistance in many cancer cells, including neuroblastoma^11^. Based on our finding that HDAC10 depletion and inhibition promoted intracellular accumulation of doxorubicin, we investigated if HDAC6/10 inhibitors interfered with P-glycoprotein function. Flow cytometric analyses proved that P-glycoprotein was present at the plasma membrane of BE(2)-C cells (Fig. 4a). Thus, we investigated whether doxorubicin accumulation after HDAC10 inhibition was P-glycoprotein dependent, using RNAi-mediated P-glycoprotein knockdown (siP-gp). Effectiveness of P-glycoprotein knockdown was shown at the cell surface (Fig. 4a,b), in whole protein lysates and with real-time RT-PCR (Supplementary Fig. S3a,b). P-glycoprotein knockdown increased the basal levels of doxorubicin in BE(2)-C cells, as did inhibition of P-glycoprotein with verapamil (Fig. 4c,d). Increased doxorubicin accumulation after HDAC6/10 inhibition also occurred in cells with P-glycoprotein knockdown (Fig. 4d). The relative increase in intracellular doxorubicin after HDAC6/10 inhibition in presence or absence of P-glycoprotein was unchanged when data were normalized to the respective basal doxorubicin fluorescence of siCtrl and siP-gp cells (Fig. 4e). In contrast, the increase in doxorubicin accumulation was significantly lower in siP-gp cells, when the P-glycoprotein inhibitor verapamil was used (Fig. 4e). Taken together, these data indicate that HDAC10 inhibition promotes intracellular accumulation of doxorubicin independent of P-glycoprotein.

### Depletion and inhibition of HDAC10 promote doxorubicin accumulation by inhibiting lysosomal exocytosis

**Figure 5:**
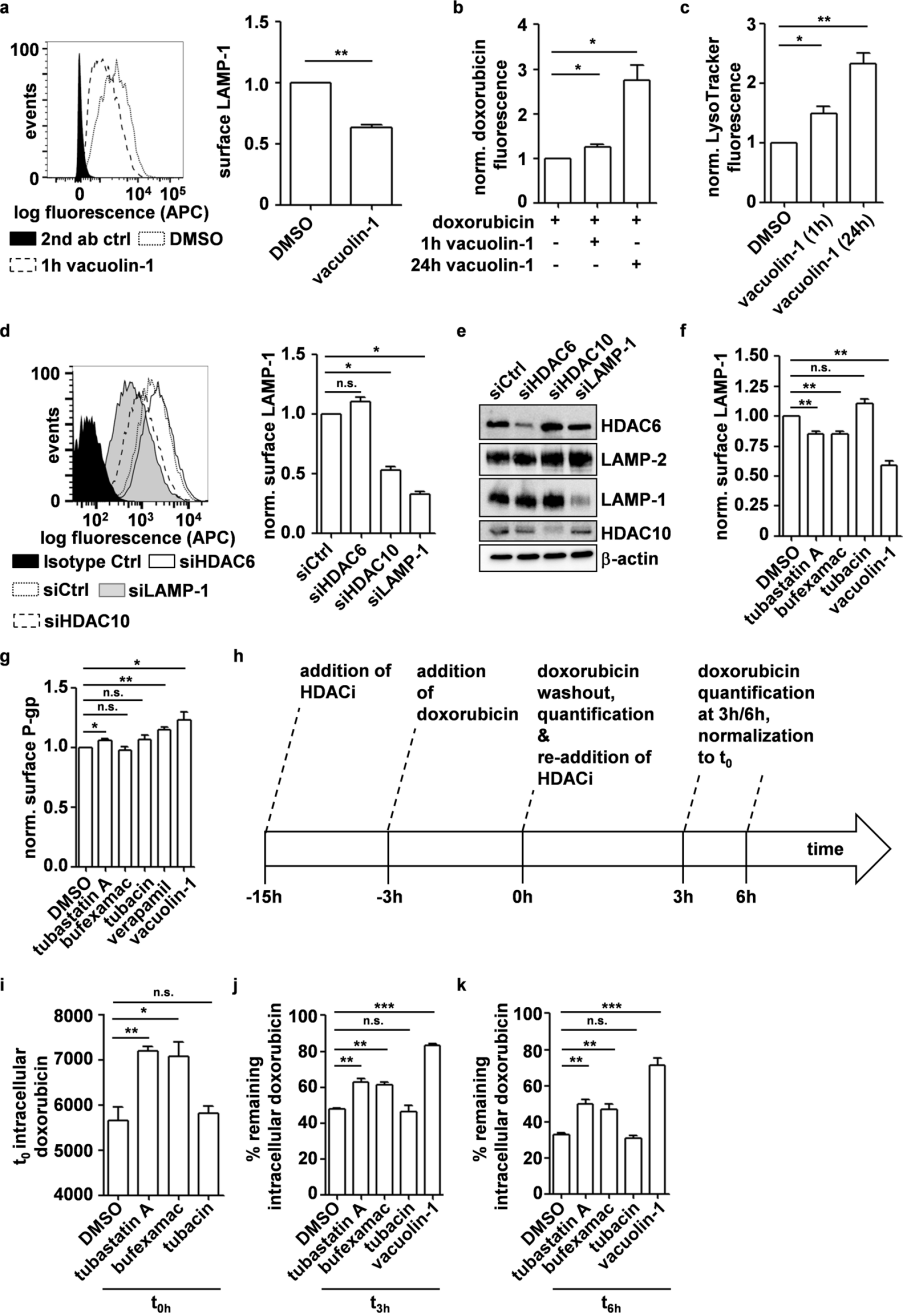
Depletion and inhibition of HDAC10 promote doxorubicin accumulation by inhibiting lysosomal exocytosis. (**a)** Flow cytometric quantification of cell surface LAMP-1 expression normalized to DMSO treated cells. Bar graph depicts mean fluorescence of n = 3 experiments. (**b)** Flow cytometric analysis of cellular doxorubicin after 48 h treatment with 100 ng/ml doxorubicin. 10 µM vacuolin-1 was added for the indicated amount of time. Bar graph depicts normalized mean fluorescence of n = 5 experiments. (**c)** Flow cytometric quantification of LysoTracker staining. Bar graph depicts mean fluorescence of at least 3 experiments normalized to DMSO treated cells. (**d)** Flow cytometric quantification of cell surface LAMP-1 expression 6d after transfection with siRNAs against HDAC6, HDAC10 and LAMP-1. Bar graph depicts mean fluorescence of n = 3 experiments normalized to siCtrl cells. (**e)** Western blot analysis of LAMP-1 expression 6d after transfection with siRNAs. LAMP-2 and LAMP-1 band are derived from two equally loaded membrane sections. Sections were separated by cutting after blotting. (**f)** Flow cytometric analysis of surface LAMP-1 expression 6 h after beginning of treatment with 7.5 µM tubastatin A, 30 µM bufexamac and 7.5 µM tubacin. Vacuolin-1 (10 µM) was added for 1 h. Bar graph depicts normalized mean fluorescence of n = 4 experiments normalized to DMSO control. (**g)** Flow cytometric quantification of cell surface P-gp expression 6 h after beginning of treatment with 7.5 µM tubastatin A, 30 µM bufexamac, 7.5 µM tubacin and 10 µM verapamil. 10 µM vacuolin-1 was added for 1 h. Bar graph depicts mean fluorescence of n = 4 experiments normalized to DMSO control. (**h**–**k**) Flow cytometric analysis of doxorubicin secretion via lysosomal exocytosis of n = 3 experiments. Cells were pre-treated with HDAC6/10 inhibitors tubastatin A (7.5 µM), bufexamac (30 µM) and HDAC6 inhibitor tubacin (7.5 µM) for 12 h (t_-15h_) and loaded with 1 µg/ml doxorubicin for 3 h at t_-3h_. Doxorubicin was then washed out (t_0_) and doxorubicin fluorescence was assessed 0 h (t_0_) (**i)**, 3 h (**j)** and 6 h (**k)** after doxorubicin removal. Bar graphs depict cellular doxorubicin fluorescence of n = 3 experiments of non-normalized data (**i)**, as well as remaining doxorubicin fluorescence in % of the fluorescence of the respective treatment condition at t_0_ (**j**,**k**). Statistical analyses were performed on non-normalized data using paired (**a**,**b**,**c**,**d**,**f**,**g**) and on data normalized to t_0_ fluorescence using unpaired (**i**–**k**), two-tailed t-test (***p < 0.001; **0.001 ≤ p < 0.01; *0.01 ≤ p < 0.05). Error bars represent SEM.

Recent publications show that lysosomes promote resistance to chemotherapeutics not only via drug sequestration but also by promoting their efflux via lysosomal exocytosis^19,21^. As shown above, interference with HDAC10 led to a marked presence of lysosomes throughout the cytoplasm, prompting us to speculate that HDAC10 might play a role in lysosomal exocytosis (Figs 1c and 2d). Also, interference with HDAC10 function increased the total intracellular accumulation of doxorubicin rather than only promoting its sequestration in lysosomes. As HDAC10 depletion and inhibition further promoted doxorubicin accumulation independent of P-glycoprotein, we investigated whether inhibition of lysosomal exocytosis was responsible for doxorubicin accumulation upon interference with HDAC10 function. The marked presence of cell surface LAMP-1 on BE(2)-C cells in the basal state suggested that these chemoresistant neuroblastoma cells use lysosomal exocytosis as a resistance mechanism (Fig. 5a). Co-treatment of cells with doxorubicin and lysosomal exocytosis inhibitor vacuolin-1^44^, which robustly reduced surface LAMP-1 while leaving total cellular LAMP-1 levels unchanged (Fig. 5a, Supplementary Fig. S3c), promoted doxorubicin accumulation in BE(2)-C cells (Fig. 5b). Vacuolin-1 treatment also increased LysoTracker staining analogous to treatment with HDAC6/10 inhibitors or knockdown of HDAC10, supporting the hypothesis that HDAC10 plays a role in lysosomal exocytosis (Figs 5c and 2c). Notably, when combining HDAC6/10 inhibitor treatment (tubastatin A) with lysosomal exocytosis inhibitor vacuolin-1, we observed no additional lysosomal accumulation compared to treatment with vacuolin-1 alone (Supplementary Fig. S4). This suggests that lysosomal accumulation upon treatment with HDAC6/10 inhibitors is likely due to lysosomal exocytosis inhibition rather than de-novo synthesis of lysosomes.

We next tested if RNAi-mediated depletion of HDAC10 affected lysosomal exocytosis levels. Knockdown of HDAC10 strongly decreased cell surface LAMP-1 levels, as did knockdown of LAMP-1. In contrast, HDAC6 knockdown slightly increased LAMP-1 expression at the cell surface (Fig. 5d), again supporting the specific function of HDAC10 within the class IIb HDAC family. Western blot analysis of whole cell lysates revealed that total LAMP-1 levels were not decreased after HDAC10 knockdown (Fig. 5e). This indicates that HDAC10 promotes lysosomal exocytosis. Treatment of BE(2)-C cells with dual specific HDAC6/10 inhibitors tubastatin A and bufexamac for 6 h likewise reduced surface LAMP-1 levels, while treatment with HDAC6 specific inhibitor tubacin did not, which corroborates our knockdown data and suggests that HDAC10 rather than HDAC6 enzymatic function promotes lysosomal exocytosis (Fig. 5f). In contrast to changes in surface presence of LAMP-1, HDAC10 inhibition did not reduce cell surface levels of P-glycoprotein. In fact, inhibition of lysosomal exocytosis with tubastatin A and vacuolin-1 rather slightly increased surface P-gp levels (Fig. 5g). Taken together, these results suggest that reduced levels of lysosomal exocytosis are responsible for the increase in cellular doxorubicin accumulation after HDAC10 knockdown and inhibition.

**Figure 6:**
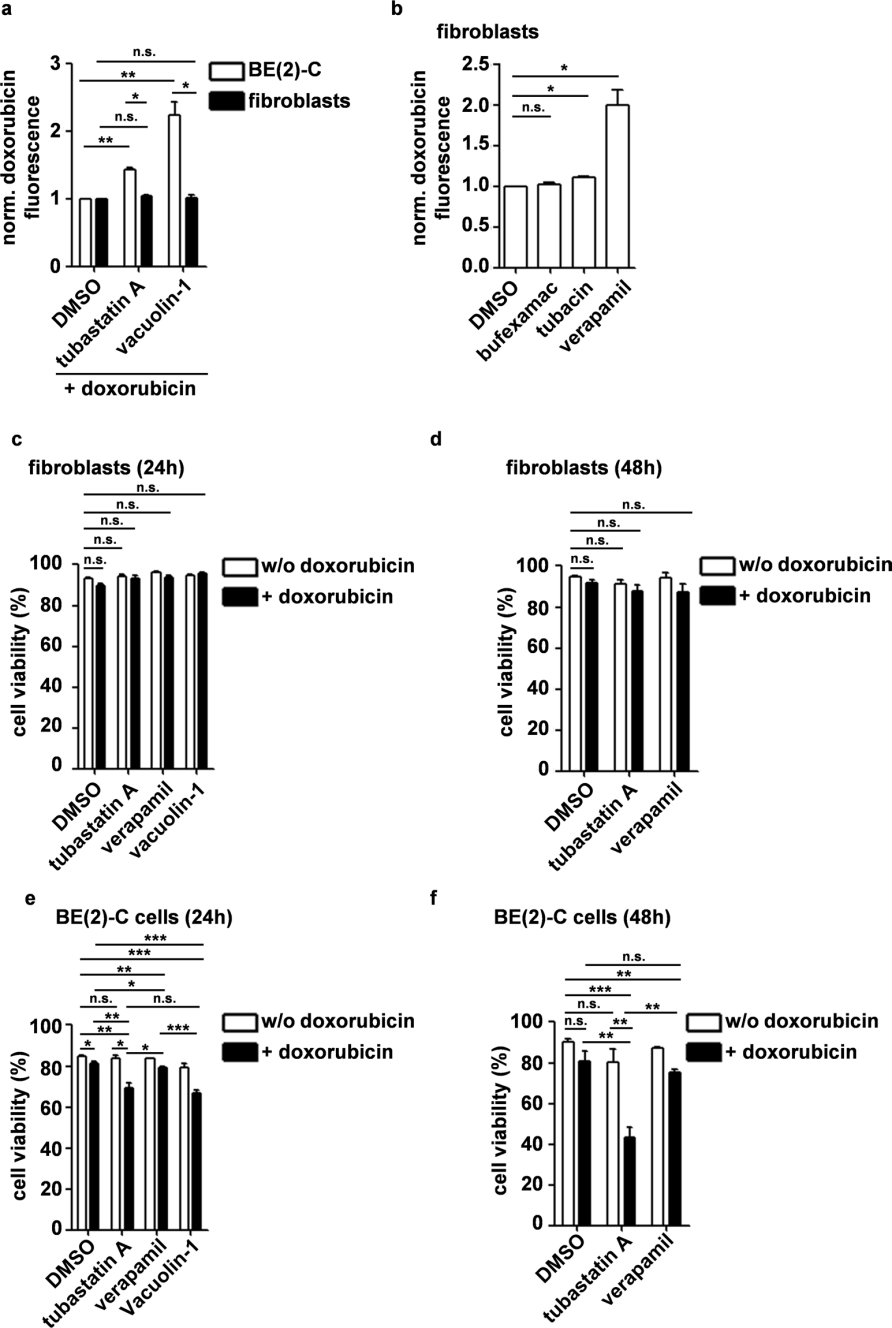
Inhibition of HDAC6/10 with tubastatin A sensitizes neuroblastoma cells but not non-malignant cells to doxorubicin treatment. (**a**) Flow cytometric analysis of cellular doxorubicin fluorescence in BE(2)-C cells and proliferating human fibroblasts 48 h after beginning of treatment with 100 ng/ml doxorubicin +/− 7.5 µM tubastatin A. Where indicated, 10 µM vacuolin-1 were added for the last 24 h. Bar graph depicts mean fluorescence of n = 3 experiments normalized to BE(2)-C cells and fibroblasts treated with doxorubicin and solvent only, respectively. (**b**) Flow cytometric analysis of cellular doxorubicin fluorescence in proliferating human fibroblasts 48 h after beginning of treatment with 100 ng/ml doxorubicin +/− 30 µM bufexamac, 7.5 µM tubacin and 10 µM verapamil, respectively. Bar graph depicts mean fluorescence of n = 3 experiments normalized to cells treated with doxorubicin and solvent only. (**c**,**d**) Viability of proliferating human fibroblasts 24 h (**c)** and 48 h (**d**) after treatment with 100 ng/ml doxorubicin +/− 7.5 µM tubastatin A, 10 µM vacuolin-1 or 10 µM verapamil (n = 3 experiments). Percentage of viable cells was assessed by automated cell counting and trypan blue exclusion. (**e**,**f**) Viability of BE(2)-C cells 24 h (**e)** and 48 h (**f)** after treatment with 100 ng/ml |doxorubicin +/− 7.5 µM tubastatin A or 10 µM verapamil (n = 3 experiments). Statistical analyses were performed on non-normalized data using paired (**a**,**b**) or unpaired (**c**,**d**,**e**,**f**), two-tailed t-test on non-normalized data (***p < 0.001; **0.001 ≤ p < 0.01; *0.01 ≤ p < 0.05). Error bars represent SEM.

To test whether HDAC10 inhibition prolonged the retention of doxorubicin, we used a pulse-chase approach in which BE(2)-C cells were pre-treated over night with HDAC6/10 inhibitors and then loaded with high concentrations of doxorubicin for 3 h (1 µg/ml). After doxorubicin washout, HDAC6/10 or HDAC6 inhibitors were re-added (t_0_), and intracellular doxorubicin fluorescence was measured at t_0_, as well as 3 h (t_3h_) and 6 h (t_6h_) after doxorubicin removal (Fig. 5h–k). Because doxorubicin was considerably increased at t_0_ in case of HDAC10 inhibition (Fig. 5i**)**, the intracellular fluorescence at t_3h_ and t_6h_ is given as a percentage of remaining intracellular doxorubicin relative to the doxorubicin fluorescence at t_0_ for each respective treatment (Fig. 5j,k). Cells treated with lysosomal exocytosis inhibitor vacuolin-1, or HDAC6/10 inhibitors bufexamac and tubastatin A retained higher levels of intracellular doxorubicin after 3h (t_3h_) (Fig. 5j) and 6h (t_6h_) (Fig. 5k), respectively. In contrast, specific HDAC6 inhibition alone by tubacin had no effect and secretion of doxorubicin was comparable to solvent control. In summary, secretion of doxorubicin was slowed down in case of HDAC6/10 inhibition but not by specific HDAC6 inhibition alone. Our data suggest that this effect is due to a reduction of lysosomal exocytosis rates.

### Inhibition of HDAC6/10 with tubastatin A sensitizes neuroblastoma cells but not non-malignant cells to doxorubicin treatment

In order to test if inhibition of lysosomal exocytosis promoted doxorubicin accumulation in non-malignant cells, we analyzed doxorubicin levels in fibroblasts after addition of HDAC10 inhibitor and vacuolin-1, respectively. Doxorubicin-treated fibroblasts, in contrast to BE(2)-C cells, did not exhibit strongly increased intracellular doxorubicin when co-treated with tubastatin A nor with vacuolin-1 (Fig. 6a). Therefore, fibroblasts might rely on other mechanisms than lysosomal exocytosis for doxorubicin secretion. In fact, only the P-glycoprotein inhibitor verapamil was able to strongly increase doxorubicin accumulation in fibroblasts (Fig. 6b). In line with this, combination treatment with 100 ng/ml doxorubicin and 7.5 µM tubastatin A for 24 h or 48 h did not induce significant cell death in proliferating fibroblasts (Fig. 6c,d). In contrast, and in line with previously published data^31^, combination treatment with 100 ng/ml doxorubicin and 7.5 µM tubastatin A for 24 h or 48 h strongly reduced viability of BE(2)-C cells (Fig. 6e,f). At the same time, treatment with 100 ng/ml doxorubicin or 7.5 µM tubastatin A alone did not substantially reduce cell viability. Combination of doxorubicin and tubastatin A was also able to effectively reduce growth of BE(2)-C cells in colony assays when cells were treated for 24 h, and the combination was substantially more effective than single treatment with doxorubicin or tubastatin A alone (Supplementary Fig. S5). In contrast to cell viability assays, which directly assess number of dead cells, colony growth can be affected by cytostatic drugs, which is reflected by our finding that single treatments also affected colony growth. Taken together with cell viability data, our findings suggest that single treatment with tubastatin A or doxorubicin has a cytostatic rather than cytotoxic effect in neuroblastoma cells, whereas combination treatment induces cell death.

Notably, combination of doxorubicin and verapamil only slightly reduced cell viability of BE(2)-C cells after 24 h when compared to doxorubicin treatment alone, and this effect was not significant at later time points (48 h) (Fig. 6e,f), even though verapamil caused substantial doxorubicin accumulation in this cell line (Fig. 4d). In contrast, combination of doxorubicin with lysosomal exocytosis inhibitor vacuolin-1 for 24 h strongly reduced cell viability, in a comparable range as the combination of doxorubicin and HDAC6/10 inhibitor tubastatin A. Treatment with vacuolin-1 alone had no substantial effect. We therefore hypothesized that HDAC10 inhibitor mediated intracellular doxorubicin accumulation per se was not sufficient to induce cell death and that additional mechanisms are required to efficiently induce cell death, one of them possibly being the inhibition of lysosomal exocytosis under stress conditions.

### HDAC10 inhibition causes double-strand breaks (DSBs) and increases DNA damage caused by doxorubicin treatment

**Figure 7:**
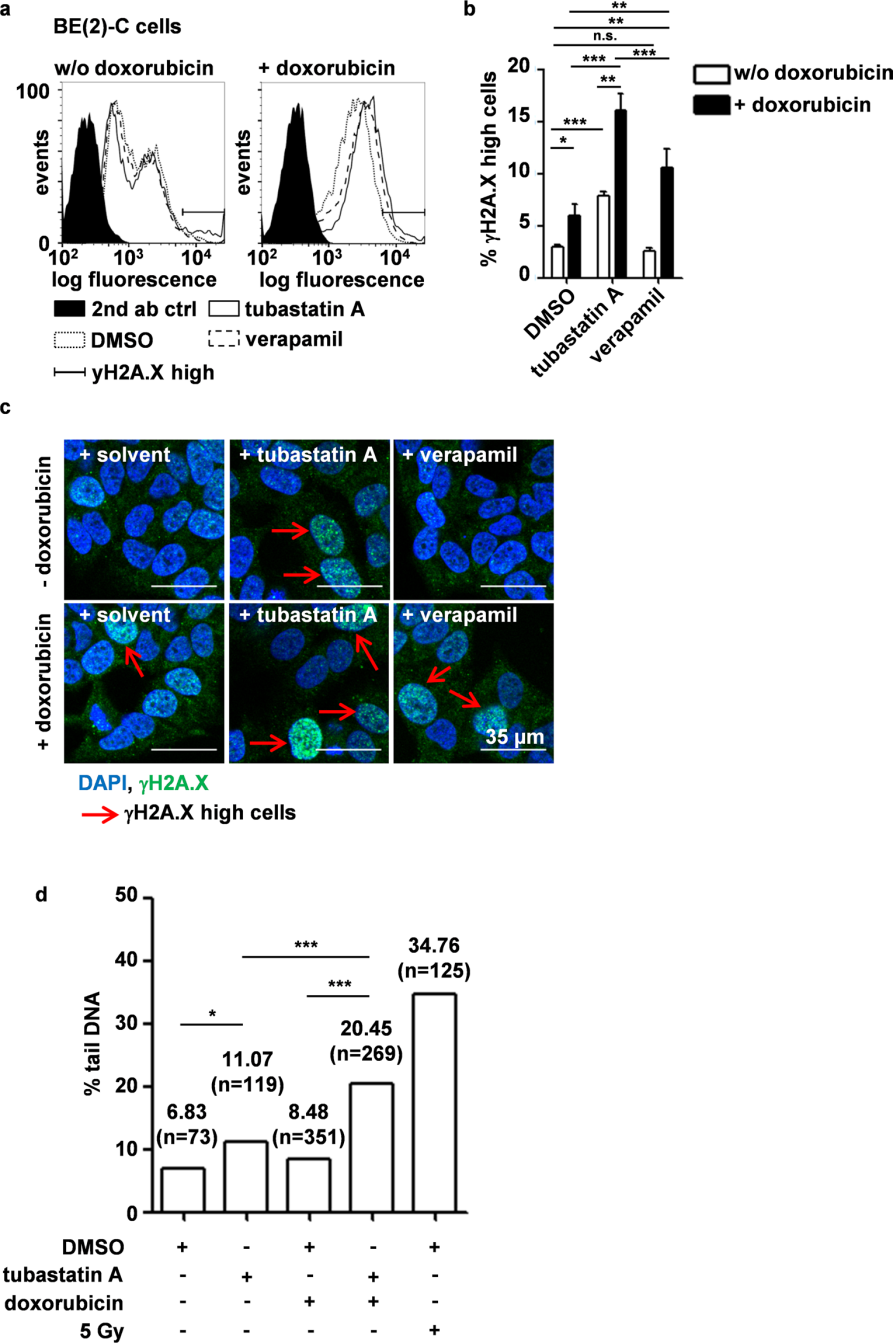
HDAC10 inhibition causes double-strand breaks and increases DNA damage caused by doxorubicin treatment. (**a**) Flow cytometric analysis of H2A.X S134 phosphorylation (γH2A.X) on fixed BE(2)-C cells treated for 24 h with 7.5 µM tubastatin A, 10 µM verapamil in the absence (left panel) or presence (right panel) of 100 ng/ml doxorubicin. Figure shows histogram of logarithmic fluorescence (x-axis) versus event number (y-axis). Ranged gates represent γH2A.X highly positive cells. (**b**) γH2A.X fluorescence of n = 6 experiments was quantified. Bar graph depicts percentage of γH2A.X highly positive cells (see (**a)**). Statistical analysis was performed using paired, two-tailed t-test on non-normalized data (***p < 0.001; **0.001 ≤ p < 0.01; *0.01 ≤ p < 0.05). Error bars represent SEM. (**c)** Immunofluorescence analysis of γH2A.X staining on fixed BE(2)-C cells treated for 24 h with 7.5 µM tubastatin A, 10 µM verapamil in the absence (left panel) or presence (right panel) of 25 ng/ml doxorubicin. Red arrows indicate nuclei with high number of DNA double-strand breaks (γH2A.X high cells). (**d)** Analysis of DSBs via comet assay. BE(2)-C cells were treated for 18 h with HDAC6/10 inhibitor tubastatin A (7.5 µM) alone or in combination with doxorubicin (100 ng/ml doxorubicin). Cells irradiated with 5 Gy (^137^Cs radiation source, dose rate of 1 Gy min^−1^) were used as positive control. Bar graph depicts median % tail DNA (y-axis) in each treatment condition of one representative experiment. Numbers above bars indicate % tail DNA and number of analyzed nuclei in brackets. Statistical analysis of two differently treated samples was performed using one-tailed Mann Whitney test.

Recent studies have shown that HDAC10 plays an important role in both DNA mismatch and DNA double-strand break repair^33,34,45^. Doxorubicin, a DNA-intercalating agent, partially causes DNA damage by topoisomerase II poisoning, which results in the occurrence of DNA double-strand breaks (DSBs)^46,47^. We, therefore, investigated whether inhibition of HDAC10 increased doxorubicin induced DSBs using flow cytometric analysis of H2A.X phospho-S134 (γH2A.X) staining. Co-treatment of BE(2)-C cells with doxorubicin and tubastatin A significantly increased the amount of DSBs compared to doxorubicin alone (Fig. 7a,b, Supplementary Fig. S6a). Co-treatment with tubastatin A also increased DSBs to a significantly greater extent than co-treatment with verapamil (Fig. 7b). In fact, treatment with tubastatin A alone was able to increase DSBs in BE(2)-C cells while treatment with verapamil did not induce DSBs in the absence of doxorubicin. (Fig. 7a,b). This was also true for treatment with lysosomal exocytosis inhibitor vacuolin-1, which did not induce DSBs on its own but increased doxorubicin induced DSBs, likely via increased intracellular doxorubicin accumulation (Supplementary Fig. S6b, Fig. 5b). Occurrence of DSBs upon HDAC10 inhibition was confirmed by the presence of γH2A.X foci in immunofluorescence stainings (Fig. 7c), as well as directly via two independent comet assays. Here, addition of tubastatin A to doxorubicin treatment substantially increased doxorubicin induced DSBs, confirming our γH2A.X data (Fig. 7d**;** Supplementary Fig. S6c). Single treatment with HDAC6/10 inhibitor tubastatin A and doxorubicin increased DSBs only trend-wise.

Taken together, our data indicate that inhibition of HDAC10 promotes intracellular accumulation of doxorubicin via lysosomal exocytosis inhibition and further promotes sensitization to chemotherapy by (i) inhibiting lysosomal exocytosis under stress conditions and (ii) inducing DSBs.

## Discussion

Resistance to anti-cancer therapy is a major issue in high-risk neuroblastoma patients. Despite the application of intense multimodal therapy, long-term prognosis in these patients remains poor^2,48^. Currently, novel experimental cancer therapy strategies aim to inhibit specific molecular targets that are important for cancer growth, survival and progression.

Given their involvement in numerous cancer-relevant pathways and their good druggability, HDACis have been developed as targeted therapies in various cancer entities, including neuroblastoma (reviewed in^49^). Indeed, a number of pan and broad-spectrum HDACis have shown efficacy in the treatment of leukemias and lymphomas and are already approved for clinical application (reviewed in^50^) or undergoing further clinical trials^51^ (NCT01422499). The use of broad-spectrum HDACis, however, is associated with dose-limiting side effects and selective inhibition of tumor-relevant HDAC isozymes could be a strategy to overcome unwanted toxicity while retaining on-target efficacy^49,52^. We have shown before that amongst other HDACs, HDAC10 plays a pivotal role in neuroblastoma chemoresistance and inhibition of HDAC10 displayed promising anti-tumor effects^31,35,53^. Other studies further indicate that HDAC10 is a potential therapeutic target in ovarian cancer^33,45^. However, the cellular functions and precise mechanisms by which HDAC10 inhibitors exert their anti-tumor effects are not completely understood.

Here, we further investigated the role of HDAC10 in lysosome-related mechanisms, as it is becoming increasingly clear that lysosomes play an important role in cancer multidrug resistance (MDR). Firstly, lysosomes contribute to chemoresistance via lysosomal sequestration or trapping, in which weakly basic drugs such as doxorubicin become trapped in the acidic lumen of lysosomes and are therefore unable to reach their target site^14–16^ (reviewed in^17,18^). Lysosomes can, in addition to ATP-driven drug efflux pumps, also contribute to drug efflux via a process known as lysosomal exocytosis, where lysosomes fuse with the plasma membrane, releasing their content to the extracellular space^19–21^ (reviewed in^22,23^). Finally, lysosomes promote cancer progression via the secretion of lysosomal hydrolases such as cathepsins, which are capable of degrading extracellular matrix proteins, thereby enhancing cancer cell invasiveness^19,21,54^. Here, we demonstrate that HDAC10 is important for lysosomal exocytosis in neuroblastoma cell lines and that chemoresistant neuroblastoma cells use this mechanism to dispose of chemotherapeutics such as doxorubicin. Interference with HDAC10 function leads to an accumulation of doxorubicin in cells independent of the drug efflux pump P-gp. These results are supported by recent evidence suggesting that lysosomes play a major role in neuroblastoma drug resistance^15^. Accumulation of doxorubicin is also achieved by co-treatment with the pan-HDAC inhibitor abexinostat, which covers HDAC10 in its inhibitory profile and is currently evaluated in phase I/II trials for combination treatment with doxorubicin^42,43^. HDAC10 inhibition does not enhance the trapping of doxorubicin, but also leads to accumulation of doxorubicin in the nucleus. Our pulse-chase data indicate that interference with HDAC10 inhibits doxorubicin efflux via lysosomal exocytosis. In this regard, lysosomal accumulation of weakly basic chemotherapeutics itself has the potential to induce lysosomal exocytosis^21^, but this was beyond the scope of our study. Recent studies further demonstrate that interfering with lysosomal function via alkalinization^55^, lysosomal permeabilization^56,57^ or lysosomal photodestruction^58^ overcomes lysosome-mediated chemoresistance in a diversity of cancer cell lines. Our data further suggest that lysosomal exocytosis could be vital for cell survival under stress conditions such as treatment with cytostatic agents.

Notably, we find no increased doxorubicin accumulation after HDAC10 inhibition in fibroblasts, even though lysosomal exocytosis is known to take place in fibroblasts^59^, possibly because these cells do not depend on HDAC10-mediated lysosomal exocytosis. It is conceivable that not all cell types equally rely on lysosomal exocytosis as a way of secreting weakly basic chemotherapeutics. Instead, these cells might primarily use other mechanisms such as ATP-driven drug efflux pumps. It is tempting to speculate that cancer cells acquire the ability to pump chemotherapeutics into lysosomes as an additional resistance mechanism against these agents. Intriguingly, P-gp itself can be localized on intracellular vesicles and has been suggested to play a major role in the sequestration of chemotherapeutics within vesicular structures^60,61^.

As with its function, the downstream targets of HDAC10 are incompletely characterized. Therefore, the mechanism how HDAC10 promotes lysosomal exocytosis remains unclear. Protein downstream targets such as HSP70 family members and the MutS homolog 2 (MSH2) have been proposed as potential HDAC10 targets^31,34^. A recent study, however, suggests that HDAC10, because of its three-dimensional structure, serves as a polyamine rather than a lysine deacetylase^62^. De-acetylated polyamines have been shown to play a role in stress resistance mechanisms such as autophagy^63^. Further, the rate-limiting enzyme of polyamine synthesis ODC-1 is a direct downstream target of MYCN (and c-MYC), directly linking deregulation of polyamine metabolism to high-risk neuroblastoma, often characterized by MYCN amplification^64,65^ (reviewed in^66^). Given the inherent link between autophagy and lysosomal function, it is conceivable that polyamines could be important for lysosomal function, although a clear link between polyamines and lysosomal function has yet to be described^67^.

Increased doxorubicin accumulation after HDAC10 inhibition cannot be solely responsible for the sensitization of neuroblastoma cell lines to doxorubicin. In fact, inhibition of P-gp by verapamil also leads to relevant doxorubicin accumulation but fails to sensitize cells as effectively as HDAC10 inhibition or lysosomal inhibition with vacuolin-1, suggesting a pro-survival role for lysosomal exocytosis under these conditions. Moreover, in light of recent reports showing that HDAC10 is involved in DNA double-strand break (DSB) repair and given the fact that doxorubicin induces DSBs by so-called topoisomerase II poisoning^33,46,47^, we assumed that this link to DNA damage might further affect doxorubicin sensitivity of neuroblastoma cells. Doxorubicin-induced DSBs are indeed strongly enhanced after tubastatin A co-treatment and tubastatin A alone is sufficient to increase DSBs in neuroblastoma cells, supporting recent studies that demonstrate a role for HDAC10 in DNA repair^33,34,45^. Co-treatment with P-gp inhibitor verapamil increases DSBs to a significantly lesser extent than tubastatin A despite promoting higher doxorubicin accumulation.

In conclusion, we demonstrate that HDAC10 inhibition sensitizes chemoresistant neuroblastoma cell lines by (i) increasing intracellular doxorubicin levels via inhibition of lysosomal exocytosis, (ii) by inhibiting pro-survival features of lysosomal exocytosis under stress conditions, and (iii) by inducing DSBs, possibly via interference with DSB repair. Thus, the combination of neuroblastoma weakly basic chemotherapeutics with selective HDAC10 inhibitors or HDAC inhibitors targeting HDAC10 in their inhibitory profile, might be beneficial for the treatment of high-risk, treatment-resistant neuroblastomas with low off-target effects on non-transformed cells.

## Methods

### Cell culture

Human neuroblastoma cell lines SK-N-BE(2)-C (hereafter referred to as BE(2)-C) (ECACC), IMR-32 (DSMZ), SK-N-AS (provided by Prof. Dr. Frank Westermann, DKFZ, Heidelberg, Germany), as well as human fibroblasts (isolated from foreskin) were cultured under standard conditions (DMEM with L-glutamine and 4.5 g/l glucose containing 10% FCS (Sigma, St. Louis, MO, USA) and 1% non-essential amino acids (NEAA; Invitrogen, Carlsbad, CA, USA)). Wild type and HDAC10-knockout HAP1 cells (Horizon Discovery Group, Cambridge, UK) were cultured in Iscove’s Modified Dulbecco’s Medium (IMDM) with 10% FCS (Sigma). All cell lines were regularly checked for contamination (Multiplexion, Heidelberg, Germany) and verified using DNA fingerprinting authentication by the DSMZ, Germany.

### Cell culture reagents and siRNAs

Bufexamac (Sigma, 100 mM stock), PCI-24781/abexinostat (Selleckchem, Houstan, TX, USA, 10 mM stock), tubastatin A (Cayman - Biomol, Hamburg, Germany, 10 mM stock), tubacin (Santa Cruz, Dallas, TX, USA, 1 mM stock), vacuolin-1 (Calbiochem, San Diego, CA, USA, 10 mM stock), valproic acid (Sigma, 1 M stock) and verapamil (Sigma, 20 mM stock) were dissolved in DMSO. MARBOSTAT-100 was dissolved in DMSO (50 mM stock). Doxorubicin (Calbiochem 504042) was bought in solution (10 mM in H_2_O). LysoTracker® Red DND-99 (1 mM stock) was purchased from ThermoFisher. Commercially available siRNAs were pooled and used for transient transfections: HDAC10 #33581 and #120681 (Ambion), HDAC6 #120451 and #120450 (Ambion), LAMP-1 #4427037 (Ambion) and P-glycoprotein SMARTpool ON-TARGETplus (L-003868-00-0005, GE Dharmacon, Lafayette, CO, USA). The NC siRNAs (Silencer Negative Control #1 and Silencer Negative Control #5; Ambion) were used as negative controls. Transfection was performed as described previously^31^ on 10 cm dishes and where indicated, cells were transferred 3–4 days after transfection into 6-well dishes for treatment.

### Western blot analysis

Western blot analysis was performed as previously described^53^. The following antibodies were used: anti-HDAC6 (sc-11420, Santa Cruz), anti-HDAC10 (H3413, Sigma), anti-acetylated histone H3 (06-911, Merck Millipore), anti-histone H3 (9715, Cell Signaling Technology, Cambridge UK), anti-LAMP-1 (H4A3, DHSB, Iowa City, IA, USA), anti-LAMP-2 (H4B4; Santa Cruz Biotechnology), anti-acetylated tubulin (6–11B-1; Sigma), anti-tubulin (2148, Cell Signaling Technology), anti P-glycoprotein (13978 S, Cell Signaling Technology) and anti-β-actin (clone AC-15; Sigma).

### Quantification of LysoTracker® Red staining via flow cytometry

Six days after siRNA transfection or 24 h after treatment with HDAC or lysosomal inhibitors, cells were stained for 1 h with 50 nM LysoTracker® Red DND-99 in medium under standard cell culture conditions. Cells were washed with ice-cold RPMI w/o phenol-red and detached using Trypsin/EDTA for 3 minutes at 37 °C. Cells were centrifuged, resuspended in RPMI w/o phenol red and LysoTracker® Red fluorescence was quantified on a BD FACSCanto II platform using the PE filter setting.

### Quantification of intracellular doxorubicin accumulation via flow cytometry

For flow cytometric quantification of intracellular doxorubicin after HDAC6/10 inhibition, BE(2)-C, SK-N-AS, IMR-32, HAP1 wild type and HDAC10 knockout cells were seeded in 6-well dishes at a density of 1.5 ∗ 10^5^ cells/well (BE(2)-C and SK-N-AS, HAP1) and 3 ∗ 10^5^ cells/well (IMR-32). Cells were treated with 25 ng/ml, 50 ng/ml, 100 ng/ml or 1 µg/ml doxorubicin, respectively, for 3–48 h (see respective figure legend). Where indicated, cells were co-treated with HDAC6/10 or lysosomal inhibitors. In case of co-treatment with vacuolin-1, vacuolin-1 was directly added to cells under doxorubicin treatment 1 h or 24 h prior to analysis. Flow cytometric quantification of intracellular doxorubicin after HDAC6, HDAC10 or P-glycoprotein knockdown in BE(2)-C cells was performed 5 days (P-glycoprotein) or 6 days (HDAC6/10) after transfection with siRNA. To that end, cells were seeded 3–4 days after transfection into 6-well dishes at 1.5 ∗ 10^5^ cells per well and treated with 100 ng/ml doxorubicin +/− HDAC6/10 and lysosomal inhibitors for the last 24 h. After doxorubicin treatment, BE(2)-C, SK-N-AS, IMR-32 and HAP1 cells were washed with ice-cold RPMI w/o phenol-red and detached using Trypsin/EDTA for 3 minutes at 37 °C. Cells were centrifuged, resuspended in RPMI w/o phenol red and doxorubicin fluorescence was quantified on a BD FACSCanto II platform using the PE filter setting.

### Fluorescence microscopic analysis of LysoTracker® Red, doxorubicin and γH2A.X staining

BE(2)-C cells were seeded at a density of 2 ∗ 10^4^ cells per well into an ibidi 8-well µ-slide one day before treatment. For analysis of doxorubicin uptake, cells were treated with 250 ng/ml doxorubicin +/− HDAC6/10 or lysosomal inhibitors for 24 h. The next day, cells were washed in PBS, fixed for 20 minutes using 4% PFA at room temperature, washed twice with PBS and counterstained with DAPI (4′,6-Diamidino-2-phenylindole). In case of LysoTracker® Red staining, cells were treated for 24 h with HDAC6/10 inhibitors. One hour before fixation, LysoTracker® Red was added at a final concentration of 50 nM and cells were stained for 1 h under standard cell culture conditions. Cells were washed as described, fixed for 15 minutes using 4% PFA at room temperature and counterstained as described above. For γH2A.X staining, cells were treated over night with 25 ng/ml doxorubicin +/− HDAC6/10 inhibitors or verapamil. Cells were fixed for 15 minutes using 4% PFA, permeabilized for 30 minutes with 0.2% Triton X-100 and blocked in 0.05% Triton X-100 and 3% BSA in PBS for 1 h at room temperature. Cells were stained with Phospho-Histone H2A.X (S139) primary antibody (CST #9718) over night (4 °C) and for 2 h with Alexa-488-labeled secondary antibody (ThermoFisher A-11008) at room temperature. Nuclei were counterstained with DAPI. Images were acquired on a Zeiss LSM710 (Oberkochen, Germany) laser scanning confocal microscope using the 40× objective.

### Flow cytometric analysis of cell surface LAMP-1 and P-glycoprotein expression and DNA double-strand breaks

Cells were dissociated from dishes using non-enzymatic cell dissociation reagent for five minutes. Cells were counted using a ViCell XR automatic cell counter (Beckmann Coulter) and 2.5 ∗ 10^5^ cells were transferred into a pre-cooled 96-well round bottom plate (TPP92097). Cells were stained for 1.5–2 hours on ice with primary anti P-gp (Biozol ABA-AB00143-1.1), LAMP-1 (H4A3, DHSB) antibody or isotype control (DLN-05794, Dianova, Barcelona, Spain) and for 1 hour with APC-labeled secondary antibody (Dianova 115-136-068). Cells were washed three times in FACS buffer (5% FBS in PBS) after each antibody incubation step. For evaluation of DNA double-strand breaks, cells were detached from dishes using trypsin, counted using a ViCell XR automatic cell counter (Beckmann Coulter) and transferred into a pre-cooled 96-well round bottom plate (4 ∗ 10^6^ cells/well). Cells were fixed and permeabilized using the eBioscience^TM^ Foxp3/Transcription Factor Staining Buffer Set (Thermo Fisher Scientific). Cells were stained for 1.5 h with Phospho-Histone H2A.X (S139) primary antibody (CST #9718) and for 1 h in Alexa-488-labeled secondary antibody (ThermoFisher A-11008). Cells were washed three times in 1× permeabilization buffer after each antibody incubation step and fluorescence was quantified on a BD FACSCanto II platform.

### Cell viability assays

Adherent cell lines were trypsinized and cells were pooled with corresponding supernatant, centrifuged and resuspended in 1.5 ml cell culture media. Cell viability was measured by automated trypan blue staining with the Vi-Cell XR Cell Viability Analyzer from Beckman Coulter (Krefeld, Germany).

### NanoBRET Assay

HeLa cells, stabily transfected with NanoBRET plasmids NanoLuc®-HDAC6 FL Fusion Vector and NanoLuc®-HDAC10 FL Fusion Vector (Promega, Madison, WI, USA) were seeded at 20.000 cells/well in white 96 well plates. Without further incubation, tracer (0.3 µM) and drugs were added in separate steps and plates were placed in a tissue culture incubator for 2 h. For NanoBRET quantification, plates were put at room temperature for 10 minutes. Nanoglow substrate, diluted in OptiMEM without phenol red, was added and measured within 10 minutes in an OPTIMA plate reader (460 nm emission for donor and 610LP filter for acceptor). The BRET signal was calculated by the ratio of acceptor signal and donor signal.

### Alkaline single cell gel electrophoresis assay (comet assay)

BE(2)-C cells were treated with the HDAC6/10 inhibitor tubastatin A alone or in combination with the genotoxic drug doxorubicin (100 ng/ml doxorubicin, 7.5 µM tubastatin) for 18 h. Untreated cell aliquots and cells irradiated with 5 Gy (^137^Cs radiation source, dose rate of 1 Gy min^−1^) were used as negative and positive controls, respectively. Cells were subsequently analyzed by the alkaline comet assay as previously described^68,69^ with some modifications. In brief, the cells were mixed with 0.7% low-melting temperature agarose (Biozym), plated on two-well slides (Trevigen), and subsequently lysed overnight. Single cell electrophoresis was performed at 4 °C. Analysis of DNA damage was accomplished by fluorescence microscopy using a fully automated cell scanning system Metafer-4 (Metasystems) as described in^70^. DNA damage was assessed as “Tail DNA in %”. Statistical evaluation of two differently treated cell aliquots was performed with the Mann Whitney test using GraphPad Prism version 5.04 for Windows.

### Statistics

All cell culture experiments were performed at least three times. In order to compare treatment groups of cell culture experiments, we performed two-tailed t test on non-normalized raw data (see respective figure legend). P values of less than 0.05 were considered as significant. IC_50_/EC_50_ values were calculated with GraphPad Prism version 5.00 for Windows, GraphPad Software, San Diego California USA.

### Data availability

Raw data used in this study are available from corresponding author upon reasonable request

## Electronic supplementary material

Supplementary Material
