# Supplementary material for: Dual role of HDAC10 in lysosomal exocytosis and DNA repair promotes neuroblastoma chemoresistance
Source: Sci Rep. 2018 Jul 3;8:10039. doi: 10.1038/s41598-018-28265-5 (PMC6030077; doi:10.1038/s41598-018-28265-5)
Supplement: Supplementary file 1 — Supplementary Material [file 41598_2018_28265_MOESM1_ESM.pdf]

Supplementary Material

Dual role of HDAC10 in lysosomal exocytosis and DNA repair promotes neuroblastoma chemoresistance

Johannes Ridinger[1, 2, 3], Emily Koeneke[1, 2, 4], Fiona R. Kolbinger[1, 2], Katharina Koerholz[1, 2], Siavosh Mahboobi[5], Lars Hellweg[6], Nikolas Gunkel[6], Aubry K. Miller[6], Heike Peterziel[1, 2], Peter Schmezer[7], Anne Hamacher-Brady[8], Olaf Witt[1, 2, 9] and Ina Oehme[1, 2; *]

[1]Preclinical Program, Hopp Children's Cancer Center at NCT Heidelberg (KiTZ)

[2]Clinical Cooperation Unit Pediatric Oncology, German Cancer Research Center (DKFZ), INF 280, D-69120 Heidelberg, Germany and German Cancer Research Consortium

[3]Faculty of Biosciences, University of Heidelberg, Germany

[4]University of Heidelberg, Heidelberg, Germany

[5]Institute of Pharmacy, University of Regensburg, Germany

[6]Research Group Cancer Drug Development, German Cancer Research Center, Heidelberg, Germany

[7]Division of Epigenomics and Cancer Risk Factors, German Cancer Research Center, Heidelberg, Germany

[8]Johns Hopkins University, Bloomberg School of Public Health, Baltimore, United States

[9]Department of Pediatric Oncology, Hematology and Immunology, University Hospital Heidelberg, Heidelberg, Germany

***Correspondence to:**

Ina Oehme, e-mail: i.oehme@dkfz.de, phone: 0049-6221-423388.

Supplementary Methods

RNA isolation and real-time RT-PCR

RNA was isolated from neuroblastoma cell lines using the RNeasy MiniKit (Qiagen) according to manufacturer's instructions. Real-time RT-PCR was performed as described in[1]. Data were normalized to neuroblastoma housekeeping genes *SDHA* and *HPRT*[2] and depicted as fold change relative to negative control. The following primer pairs were used: *HPRT* (hypoxanthine phosphoribosyltransferase 1, forward: 5′-TGACACTGGCAAAACAATGCA-3′ reverse: 5′-GGTCCTTTTCACCAGCAAGCT-3′, *ABCB1 (P-GP), forward: 5'-GGGATGGTCAGTGTTGATGGA-3', reverse: 5'-GCTATCGTGGTGGCAAACAATA-3'* , *SDHA* (succinate dehydrogenase complex, subunit A, forward: 5′-TGGGAACAAGAGGGCATCTG-3′, reverse: 5′-CCACCACTGCATCAAATTCATG-3′).

Colony formation assay

BE(2)-C cells were seeded on 6-well plates at a density of 800 cells per well and treated as indicated for 24 hours. After 24 hours, drugs were washed out and cells were allowed to grow for 11 more days before staining of viable cell colonies with crystal violet (1 % crystal violet (w/v) in 70% ethanol). For quantification, the plates were scanned, and colonies were counted in 16-bit binary pictures with the ITCN plugin for ImageJ software (U. S. National Institutes of Health, Bethesda, MD, USA; http://imagej.nih.gov/ij/).

Supplementary Figures

Supplementary Figure S1

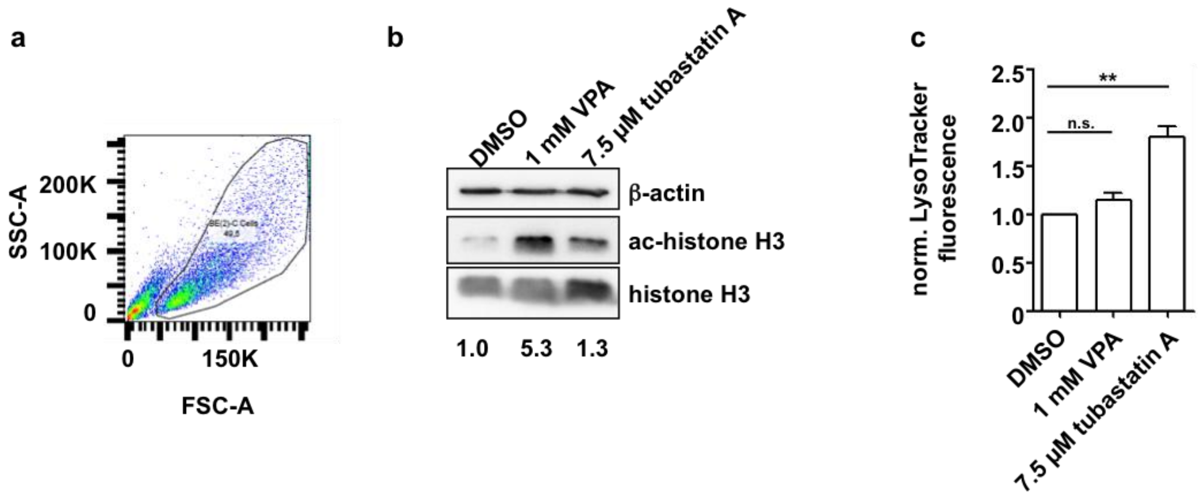

Supplementary Figure S1: Class I HDAC inhibition does not promote lysosomal accumulation in neuroblastoma cells

(a) Pseudocolor dot plot showing gating of BE(2)-C cells. **(b)** Western blot analysis of histone H3 acetylation 6h after treatment with 1 mM class I HDAC inhibitor valproic acid (VPA) and 7.5 µM class IIb HDAC inhibitor tubastatin A, respectively. Numbers below blot indicate histone H3 acetylation normalized to total histone H3 expression and relative to DMSO treated control. Bands of indicated proteins were developed on the same blot. **(c)** Flow cytometric analysis of LysoTracker DND-99 staining in BE(2)-C cells 24h after treatment with class I HDAC inhibitor VPA and class IIb HDAC inhibitor tubastatin A. Statistical analyses were performed on non-normalized data using paired, two-tailed t-test (***$p < 0.001$; **$0.001 \leq p < 0.01$; *$0.01 \leq p < 0.05$). Error bars represent SEM.

Supplementary Figure S2

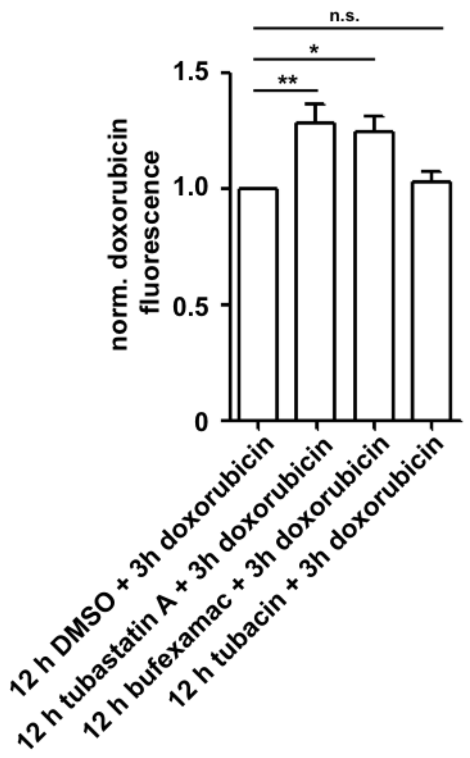

Supplementary Figure S2: Short-term HDAC6/10 but not HDAC6 inhibition promotes intracellular doxorubicin accumulation

Flow cytometric analysis of intracellular doxorubicin levels. Cells were pre-treated with DMSO, HDAC6/10 inhibitors tubastatin A (7.5 µM), bufexamac (30 µM) and HDAC6 inhibitor tubacin (7.5 µM) for 12h. Cells were then incubated for 3 more hours with 1 µg/ml doxorubicin. Bar graph depicts doxorubicin fluorescence of n=3 experiments normalized to DMSO control. Statistical analyses were performed on non-normalized data using unpaired, two-tailed t-test (***$p < 0.001$; **$0.001 \leq p < 0.01$; *$0.01 \leq p < 0.05$). Error bars represent SEM.

Supplementary Figure S3

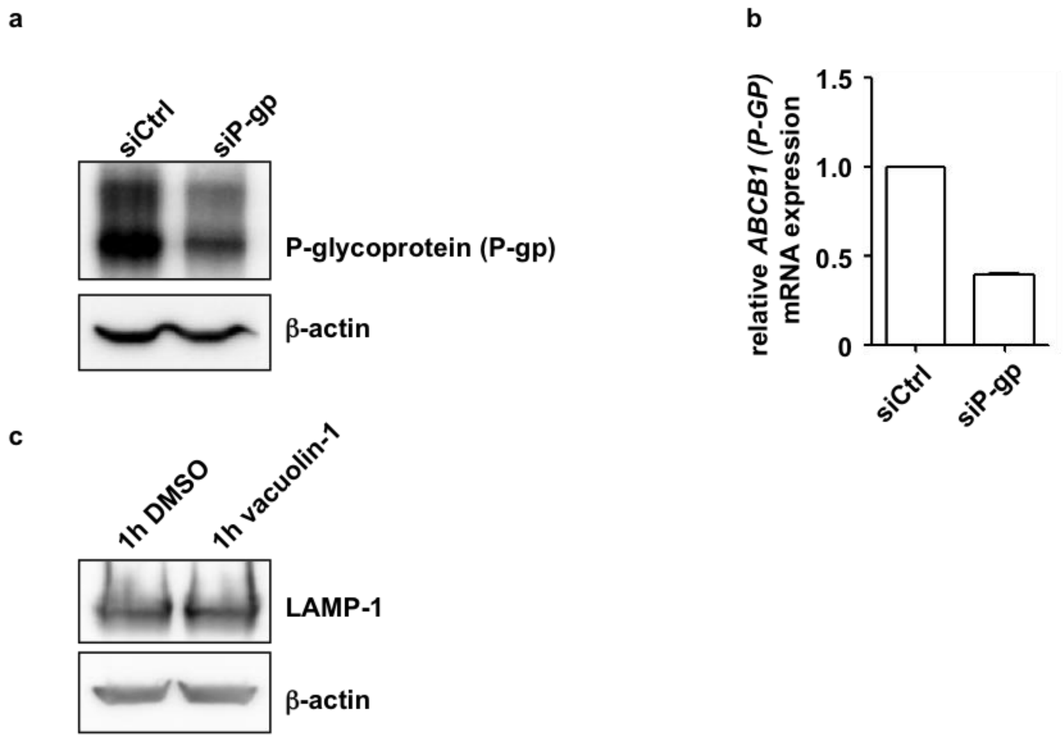

Supplementary Figure S3: Control of total P-glycoprotein (*ABCB1/P-GP)* after knockdown and total LAMP-1 levels after lysosomal exocytosis inhibition

(a) Western Blot analysis of total P-glycoprotein (P-gp) expression 5d after transfection with siRNAs against P-gp. Bands of indicated proteins were developed on the same blot. **(b)** Analysis of *ABCB1 (P-GP)* mRNA expression 5d after transfection with siRNAs against P-glycoprotein via real-time RT-PCR. Data were normalized to neuroblastoma housekeeping genes *SDHA* and *HPRT* and are depicted relative to cells transfected with control siRNAs. **(c)** Western blot analysis of total LAMP-1 expression after 1h treatment with lysosomal exocytosis inhibitor vacuolin-1 (10 µM). Bands of indicated proteins were developed on the same blot.

Supplementary Figure S4

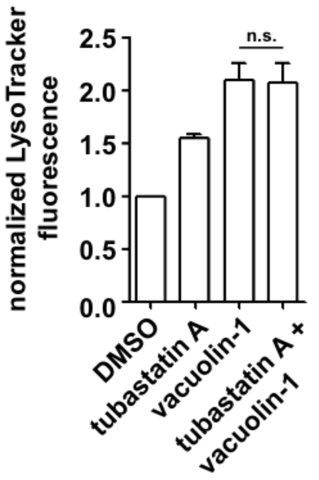

Supplementary Figure S4: HDAC6/10 inhibition does not increase lysosomal accumulation in case of lysosomal exocytosis inhibition with vacuolin-1

Flow cytometric analysis of LysoTracker DND-99 staining in BE(2)-C cells 24h after treatment with lysosomal exocytosis inhibitor vacuolin-1 (10 µM), HDAC6/10 inhibitor tubastatin A (7.5 µM) and the respective combination. Bar graph depicts mean LysoTracker fluorescence of n = 3 experiments. Statistical analyses were performed on non-normalized data using paired, two-tailed t-test (***$p < 0.001$; **$0.001 \leq p < 0.01$; *$0.01 \leq p < 0.05$). Error bars represent SEM.

Supplementary Figure S5

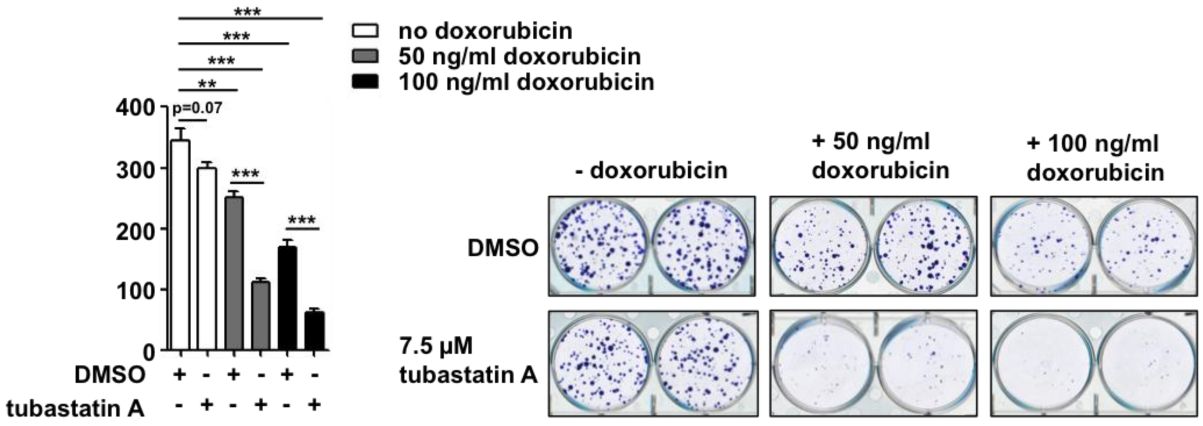

Supplementary Figure S5: Combination of doxorubicin with HDAC6/10 inhibitor tubastatin A inhibits BE(2)-C colony growth

BE(2)-C cells were treated for 24h with doxorubicin, ,tubastatin A or the combination at the respective concentrations given in the figure. Colonies were allowed to grow for eleven more days and stained with crystal violet. Bar graphs depict mean colony number. Statistical analyses were performed on non-normalized data using unpaired, two-tailed t-test (***$p < 0.001$; **$0.001 \leq p < 0.01$; *$0.01 \leq p < 0.05$). Error bars represent SEM.

Supplementary Figure S6

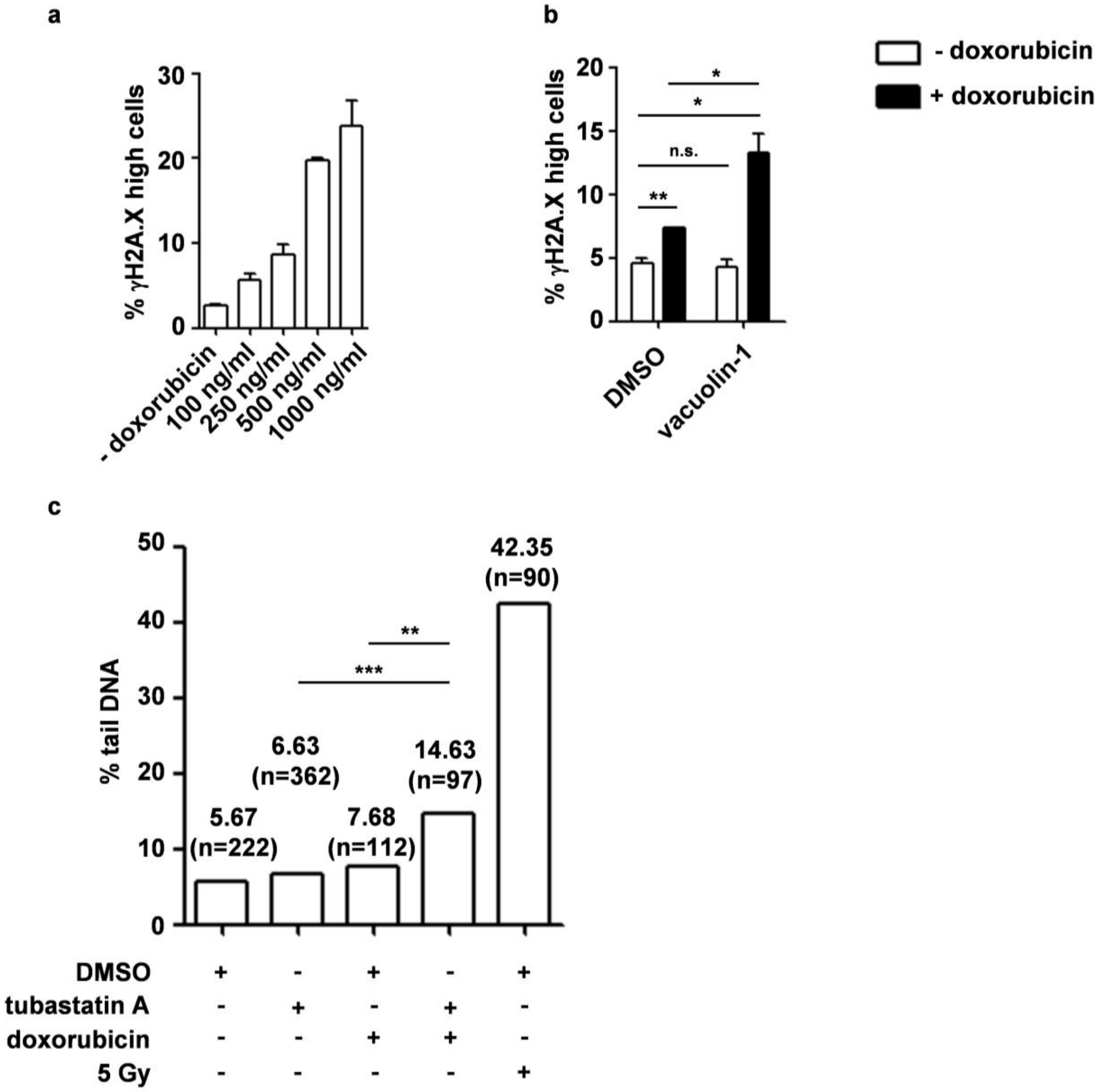

Supplementary Figure S6: Combination of doxorubicin and vacuolin-1 increases doxorubicin induced DNA double strand breaks

(a) Flow cytometric analysis of H2A.X S134 phosphorylation (γH2A.X) on fixed BE(2)-C cells treated for 24h with various doxorubicin doses (100 ng/ml, 250 ng/ml, 500 ng/ml and 1000 ng/ml). Bar graphs depict percentage of γH2A.X highly positive cells of one representative experiment. **(b)** Flow cytometric analysis of H2A.X S134 phosphorylation on BE(2)-C cells treated for 24h with 10 µM vacuolin-1 in the absence or presence of 100 ng/ml doxorubicin. Bar graphs depict percentage of

γH2A.X highly positive cells of n= 4 experiments. Statistical analysis was performed using paired, two-tailed t-test on non-normalized data (***$p < 0.001$; **$0.001 \leq p < 0.01$; *$0.01 \leq p < 0.05$). Error bars represent SEM. **(c)** Analysis of DSBs via comet assay. BE(2)-C cells were treated for 18h with HDAC6/10 inhibitor tubastatin A (7.5 µM) alone or in combination with doxorubicin (100 ng/ml doxorubicin). Cells irradiated with 5 Gy (^{137}Cs radiation source, dose rate of 1 Gy min-^{1}) were used as positive control. Bar graph depicts median % tail DNA (y-axis) of each treatment of one replicate experiment. Numbers above bars indicate % tail DNA and number of analyzed nuclei in brackets. Statistical analysis of two differently treated samples was performed using one-tailed Mann Whitney test.

Uncropped western blots

Uncropped blots of Figure 1a

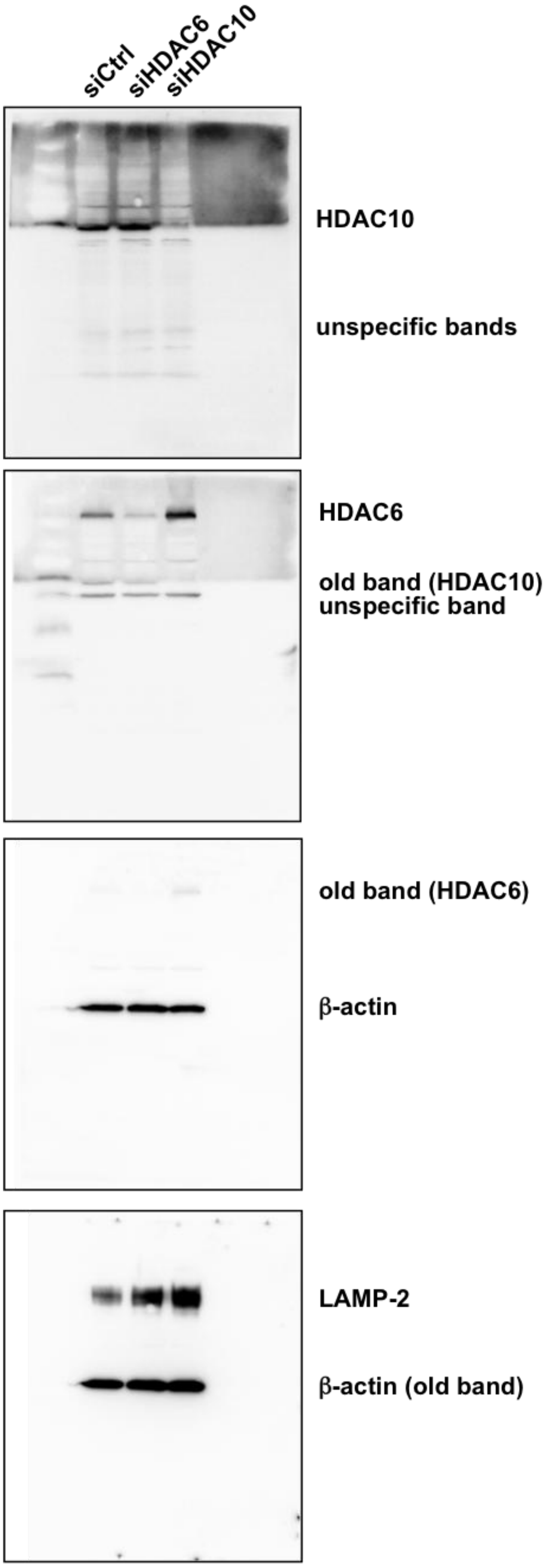

Uncropped blots of Figure 1d

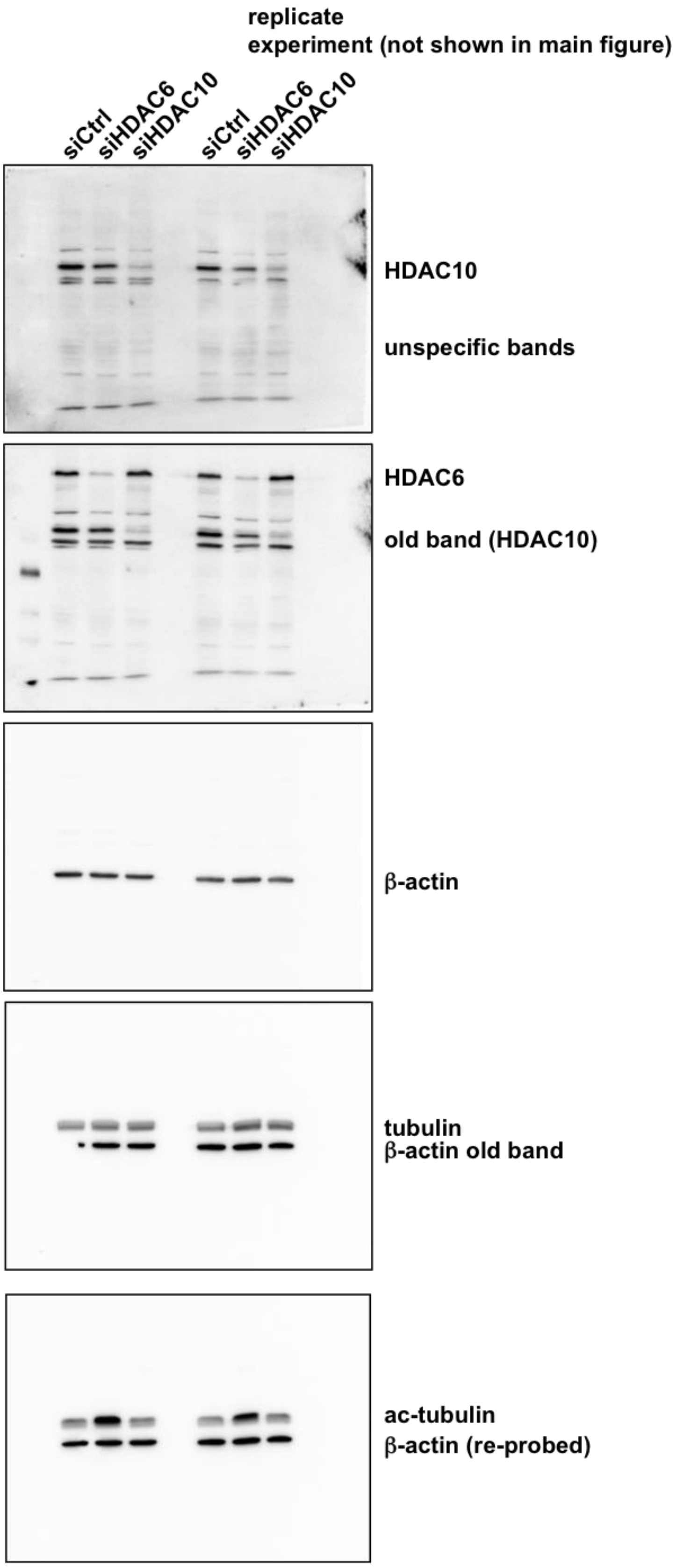

Uncropped blots of Figure 2b

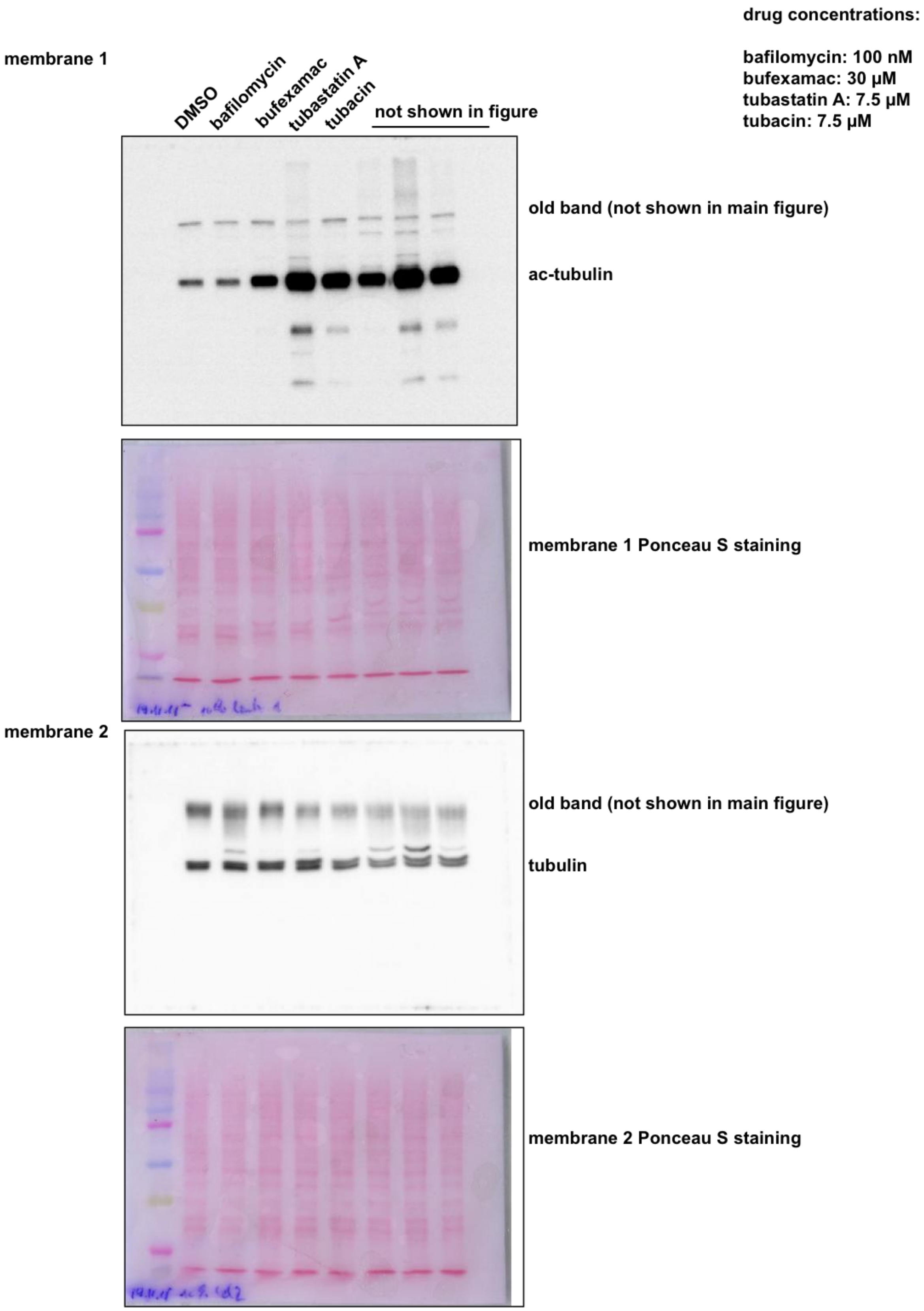

Uncropped blots of Figure 2g

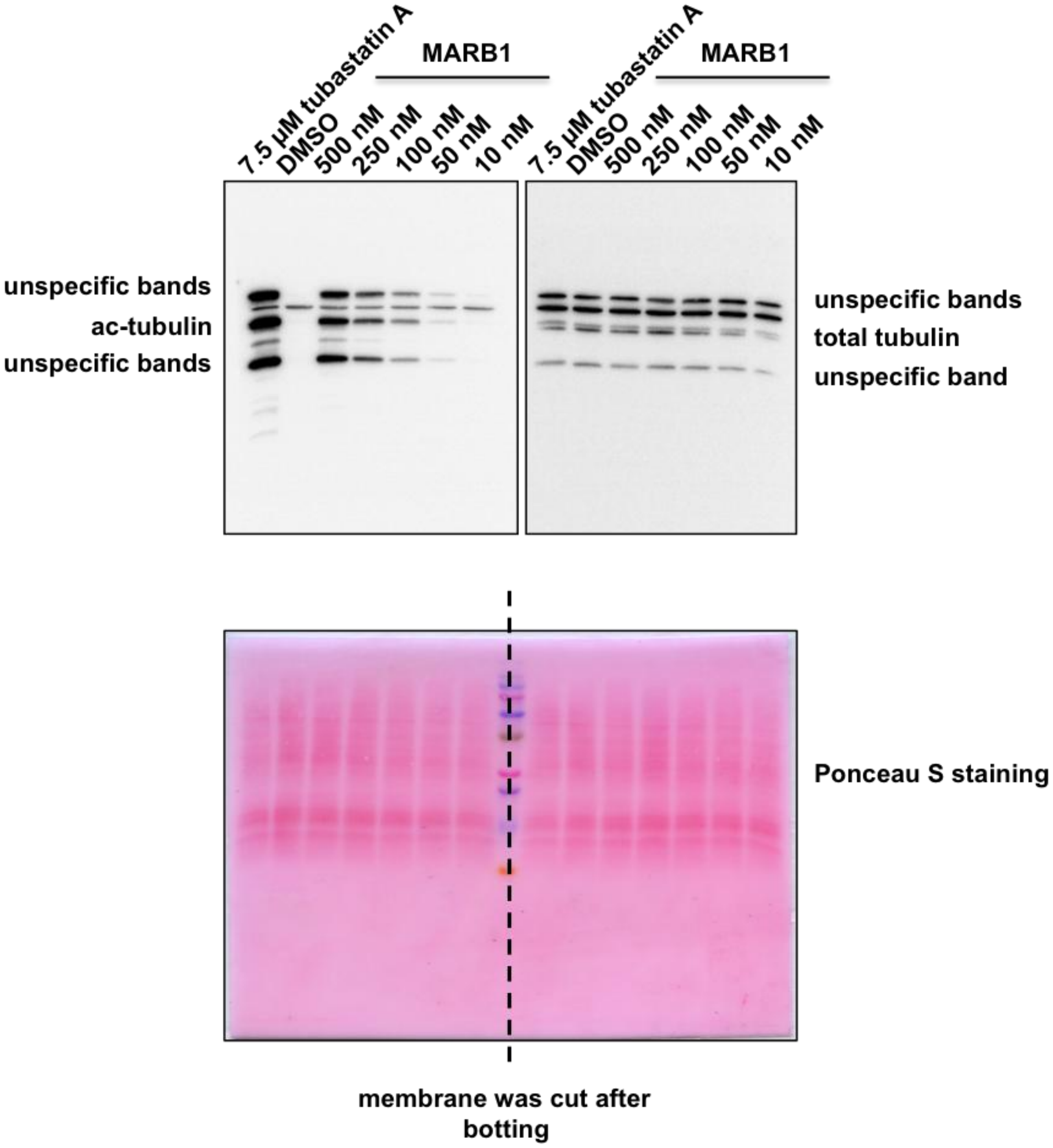

Uncropped blots of Figure 3f

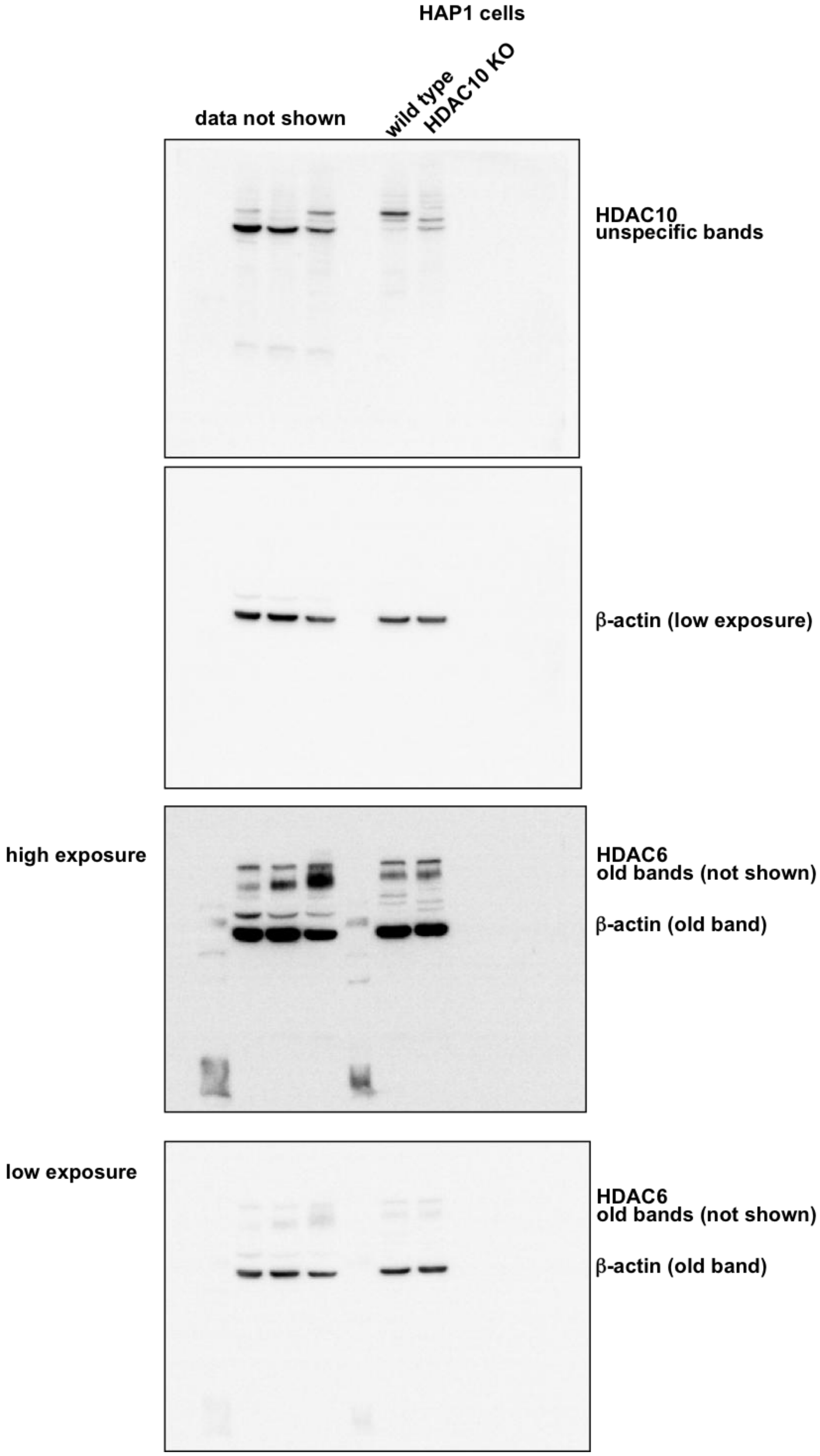

Uncropped blots of Figure 5e

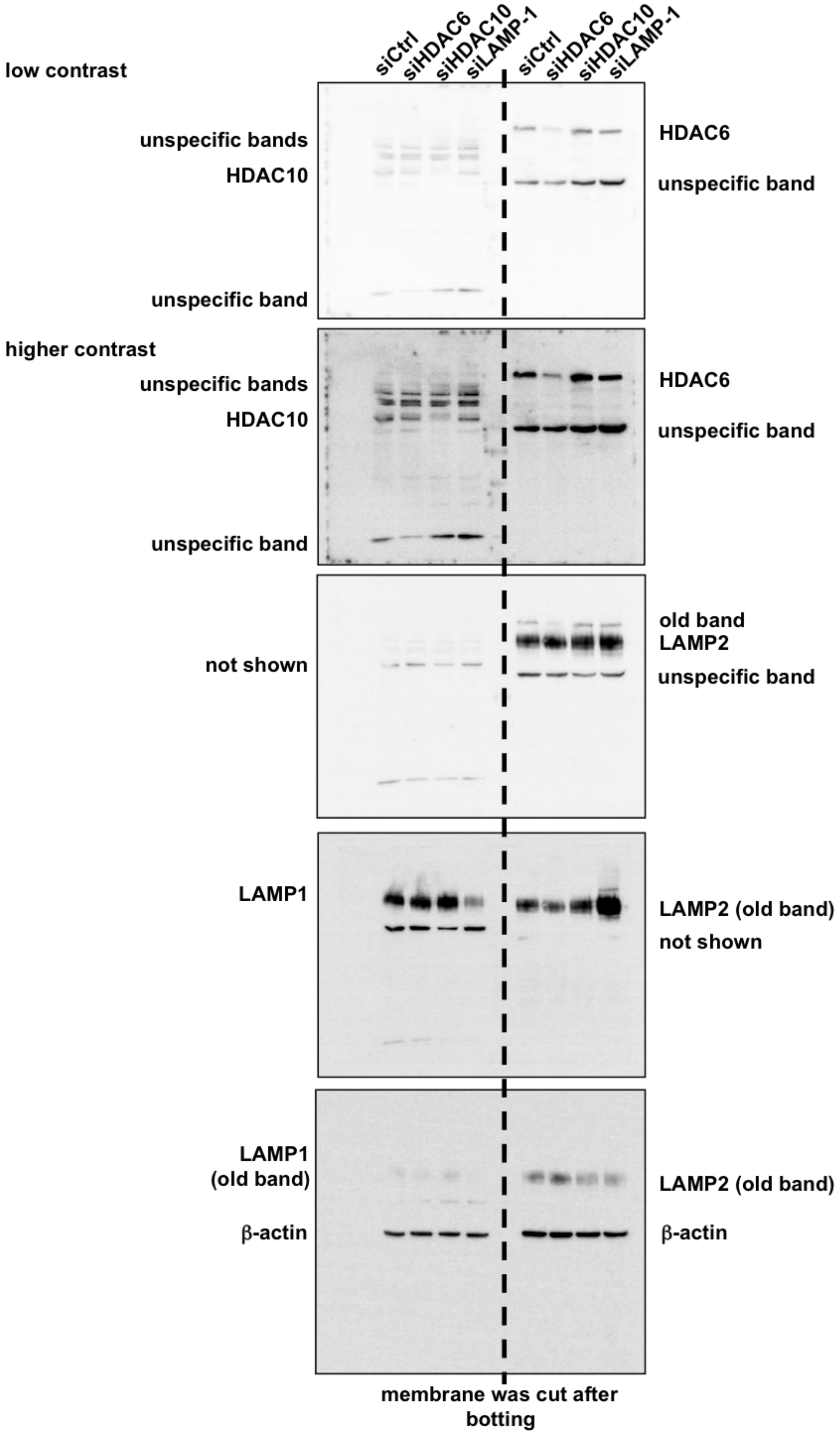

Uncropped blots of Supplementary Figure S1b

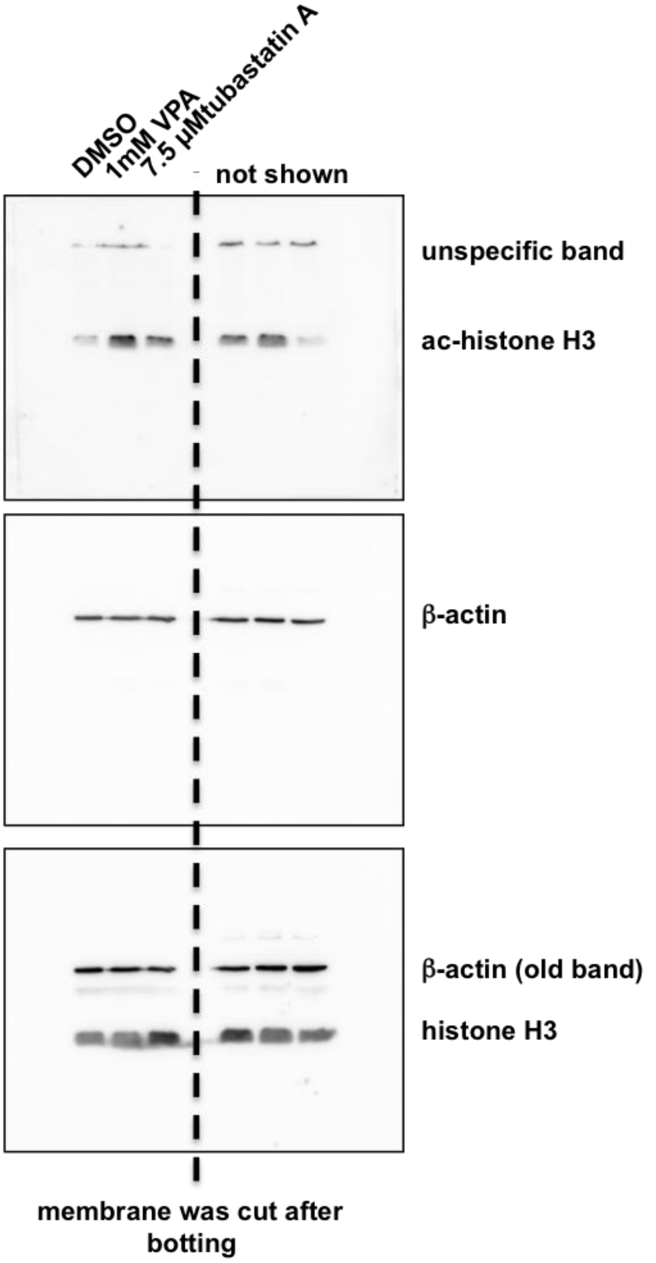

Uncropped blots of Supplementary Figure S3a

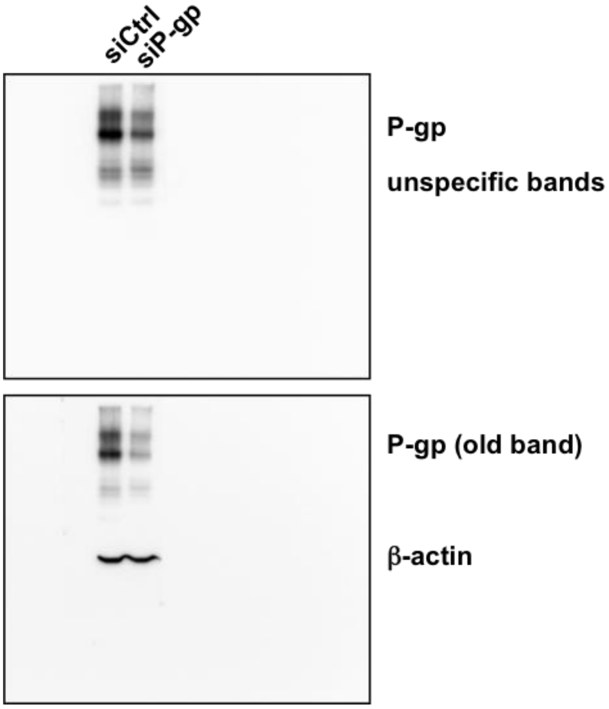

Uncropped blots of Supplementary Figure S3c

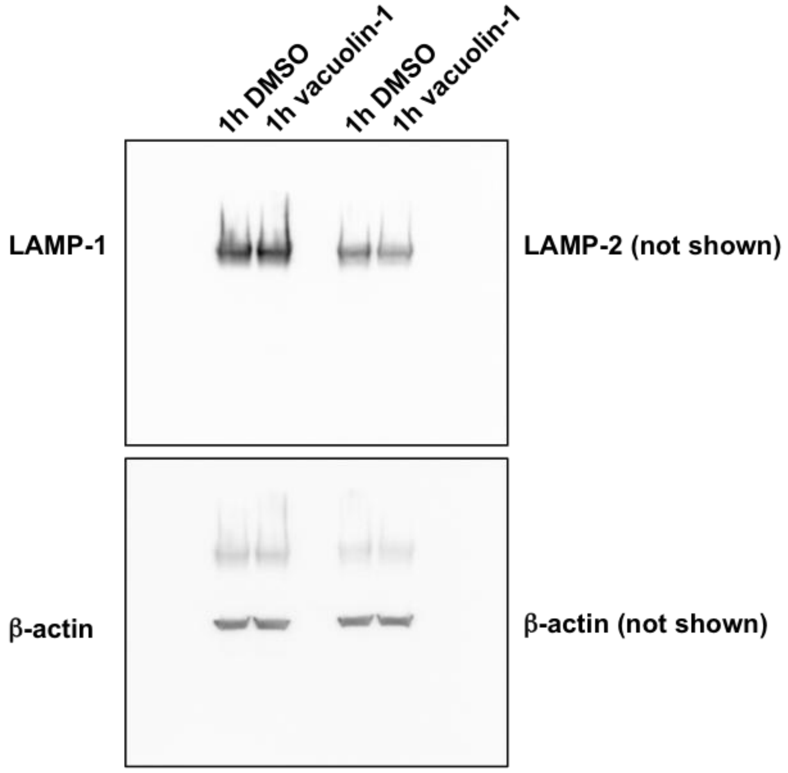

Supplementary References

1 Oehme, I. *et al.* Histone deacetylase 8 in neuroblastoma tumorigenesis. *Clin Cancer Res* 15, 91-99, doi:10.1158/1078-0432.CCR-08-0684 (2009).

2 Fischer, M., Skowron, M. & Berthold, F. Reliable transcript quantification by real-time reverse transcriptase-polymerase chain reaction in primary neuroblastoma using normalization to averaged expression levels of the control genes HPRT1 and SDHA. *J Mol Diagn* 7, 89-96, doi:10.1016/S1525-1578(10)60013-X (2005).
